# Identifying the Morphological and Molecular Features of a Cell-Based Orthotopic Pancreatic Cancer Mouse Model during Growth over Time

**DOI:** 10.3390/ijms25115619

**Published:** 2024-05-22

**Authors:** Felista L. Tansi, Andrea Schrepper, Michael Schwarzer, Ulf Teichgräber, Ingrid Hilger

**Affiliations:** 1 Experimental Radiology, Institute of Diagnostic and Interventional Radiology, Jena University Hospital, Friedrich Schiller University Jena, Am Klinikum 1, 07747 Jena, Germany; 2 Department of Cardiothoracic Surgery, Jena University Hospital, Friedrich Schiller University Jena, Am Klinikum 1, 07747 Jena, Germanymichael.schwarzer@med.uni-jena.de (M.S.); 3 Institute of Diagnostic and Interventional Radiology, Jena University Hospital, Friedrich Schiller University Jena, Am Klinikum 1, 07747 Jena, Germany

**Keywords:** pancreatic cancer, tumor stroma, extracellular matrix, orthotopic cancer models, tumor progression, ultrasound imaging, fluorescence imaging, pancreas, omentum, pancreatic stellate cells, alpha-smooth muscle actin (αSMA), fibroblast activation protein (FAP), collagen fiber

## Abstract

Pancreatic ductal adenocarcinoma (PDAC), characterized by hypovascularity, hypoxia, and desmoplastic stroma is one of the deadliest malignancies in humans, with a 5-year survival rate of only 7%. The anatomical location of the pancreas and lack of symptoms in patients with early onset of disease accounts for late diagnosis. Consequently, 85% of patients present with non-resectable, locally advanced, or advanced metastatic disease at diagnosis and rely on alternative therapies such as chemotherapy, immunotherapy, and others. The response to these therapies highly depends on the stage of disease at the start of therapy. It is, therefore, vital to consider the stages of PDAC models in preclinical studies when testing new therapeutics and treatment modalities. We report a standardized induction of cell-based orthotopic pancreatic cancer models in mice and the identification of vital features of their progression by ultrasound imaging and histological analysis of the level of pancreatic stellate cells, mature fibroblasts, and collagen. The results highlight that early-stage primary tumors are secluded in the pancreas and advance towards infiltrating the omentum at week 5–7 post implantation of the BxPC-3 and Panc-1 models investigated. Late stages show extensive growth, the infiltration of the omentum and/or stomach wall, metastases, augmented fibroblasts, and collagen levels. The findings can serve as suggestions for defining *growth parameter-based stages* of orthotopic pancreatic cancer models for the preclinical testing of drug efficacy in the future.

## 1. Introduction

The anatomical position of the pancreas is disadvantageous for the early detection of pancreatic ductal adenocarcinoma (PDAC) in humans. The onset of symptoms, such as pain which results from the infiltration of the surrounding nerves or jaundice that results from the occlusion of the bile ducts, indicate advanced stages [1,2,3]. Though complete surgical removal is the sole hope for the cure of pancreatic adenocarcinomas, a majority of pancreatic cancers are detected late, when surgical removal poses a higher risk than benefit [4,5]. Patients with non-resectable pancreatic cancers, therefore, rely on alternative treatment options, including chemotherapy, radiotherapy, immunotherapy, and other emerging modalities [6,7]. The poor response of these cancer stages to treatment accounts for the high mortality associated with pancreatic cancer until today [5,8]. Thus, continuous efforts in the design of new treatment options and their initial preclinical testing are inevitable for the future management of pancreatic cancer.

Xenograft models are irreplaceable for such preclinical studies. They permit a rapid and repeated analysis of disease pathogenesis, the testing of new therapeutic options, as well as the efficacy of novel therapeutic drugs [9]. Although genetically engineered mice models of orthotopic pancreatic tumors have the advantage of spontaneous occurrence and growth similar to the incidence in humans [10], repeated drug testing in a large number of animals bearing such orthotopic models is time consuming and limited by a low number of animals with ascertained tumor growth. Furthermore, the tumor cell properties of genetically engineered orthotopic murine models are of the host origin, and the progression to late stages as in humans is limited by the comparably short life span of the mouse [9]. For decades, researchers have, therefore, used heterotopic and orthotopic murine models of continuously propagated tumor cell lines established from human carcinomas or grafted human tumor fragments. Thereby, the human tumor cells are implanted subcutaneously on different parts of the mouse body or orthotopically into the corresponding organ of the tumor cell origin [9,11,12]. The use of heterotopic tumor models remains valuable for rapid preclinical studies. However, increasing research observations have raised awareness of various differences that exist between heterotopic and orthotopic models. For example, the RNA and protein synthesis [13], metabolism, such as lipid processing [14], as well as the response to therapeutic drugs [15] varies between heterotopic and orthotopic pancreatic cancer models in mice. The implanted human tumor acquires its microenvironment from the host, including certain features from its immediate surrounding [16,17]. These include the extracellular matrix, pancreatic stellate cells, mature fibroblasts, immune cells, and vasculature, which all stem from the host mouse. Only immune-compromised mice tolerate the tumor growth of implanted cells or grafts, and hence, the tumors possess comparably limited immunity. However, these models still convey benefits that outweigh the cost, time, and success limitations of genetically engineered models or those tried in immune-competent mice. Moreover, the implanted tumor cells retain their human properties and thus are suitable for diverse preclinical research questions [15,16,18,19,20], such as the validation of therapeutic efficacy.

In the clinical situation, the response of tumors to drugs depends on the tumor microenvironment and tumor stage. Conventional therapies are more efficient in the early, but not late, stages of cancers. Therapeutic options such as photothermal therapy [21], high-frequency hyperthermia [22], magnetic hyperthermia [7,23], or focal cryotherapy [24] could be promising adjuvant therapies for PDAC, especially for the locally advanced non-resectable stages. To validate their potential in animal models of PDAC, it is vital to use orthotopic tumor models and to select the “defined stages” of the tumor progression in order to draw reliable conclusions about the efficacy of the therapy in question. Enormous efforts in preclinical research and drug testing have regrettably not considered tumor staging based on molecular features [10,15,16]. We questioned if there are structural and molecular features of preclinical orthotopic pancreatic cancer mice models that reveal when the tumors are in the early or late stages of growth.

Human and mouse pancreas share some structural similarities [25]. Hence, the orthotopic models of human pancreatic cancers acquire features from the mouse pancreas during growth that mimic the progression of the tumor in humans [15,18]. Research on cell- and graft-based orthotopic PDAC models revealed that tumor growth depends on the location of the injection and the volume injected [15]. This study implemented three anatomical approaches of incision, namely the left flank incision, the midline laparotomy, and the left subcostal incision for pancreas exposure and cell injections, and showed differences in the anatomical location of the tumors that were, however, related to the accessibility of the pancreas body by the different incision approaches [15]. Moreover, the researchers compared the growth rate, drug sensitivity, and molecular differences between the orthotopic and heterotopic tumor models. Though the tumors were analyzed with respect to different molecular features, they were not classified in “parameter-based growth stages”. According to the recommendations of the study, the use of the 50% (*v*/*v*) growth factor containing Matrigel^®^ matrix solution for cell preparation and the left flank incision approach enabled easy access of the mouse pancreas and the most accurate implantations into the pancreas body (splenic lobe).

In the underlying work, we established human cell-based orthotopic tumor models in mice using a 2% growth factor reduced Matrigel^®^ matrix to minimize external influence on tumor stroma and growth and implemented the left flank incision for implantation. We used the gemcitabine-resistant [26] human PDAC cell lines Panc-1 [27] with a stable far-red fluorescent mkate2 label (termed Panc-1-fl) and BxPC-3 [28]. BxPC-3 contains a wild-type Kirsten rat sarcoma (*KRAS*) oncogene as seen in 10% of PDAC patients, while Panc-1 has a mutant *KRAS* [29], like in 90% of PDAC patients. *KRAS*-mutations contribute immensely in varying sensitivities to therapies and raised awareness of the relevance of including *KRAS* molecular profiling as a vital aspect of PDAC diagnosis [30]. We asked whether using the tumor morphology monitored at different time points by imaging and correlating this with molecular signatures of pancreatic stellate cells (PSC) and collagen would identify basic aspects related to the tumors developmental stage. Naïve stellate cells are rich in vitamin A, which they lose during transformation into mature fibroblasts. An increase in vitamin A deficient mature fibroblasts augments collagen metabolism, tumor growth, and desmoplasia. It has been shown that naïve pancreatic stellate cells in PDAC differentiate into two distinct populations of cancer-associated fibroblasts (CAFs), namely myofibroblastic CAF (myCAFs) and inflammatory CAFs (iCAFs) [31,32], based on phenotype, location in the stroma, and role in cancer progression and desmoplasia. While myCAFs express elevated alpha smooth muscle actin (αSMA) levels and are tumor restrictive, iCAFs have basal αSMA levels, express fibroblast activation protein (FAP), and secrete IL-6 [31]. Assessing the molecular features of fibroblasts together with the morphological changes in the tumors over growth time could reveal insights into the tumor’s growth stage.

## 2. Results

### 2.1. In a Minor Number of Mice, Implanted Tumors Remained Secluded within the Pancreas, and Almost Outgrew the Pancreas over Time

**Figure 1:**
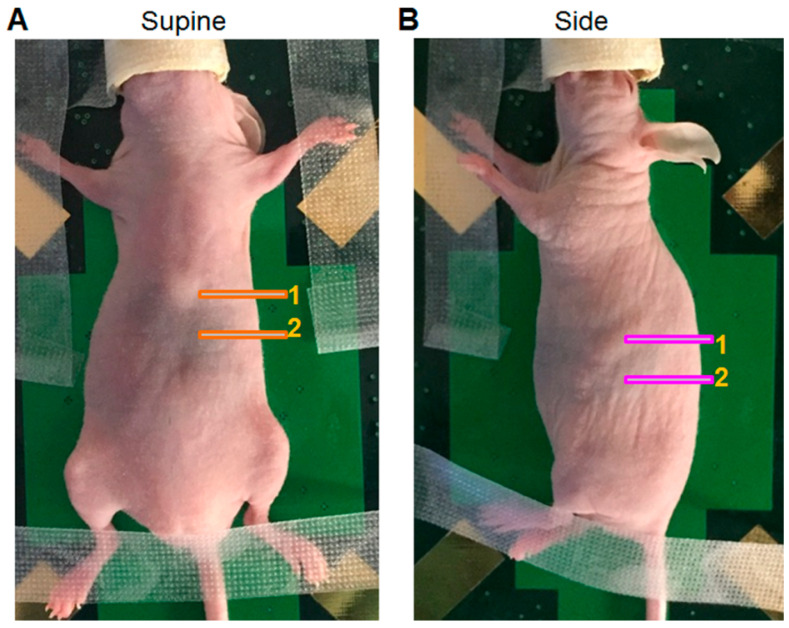
Photographic demonstration of a mouse on the stage of the ultrasound system in the supine and side positions. (**A**) The lines indicated with “1” in the supine position depicts the starting position of the scanning, whereas “2” indicates the approximate position at which a cross-section of the liver lobe, the stomach and the intersection to duodenum can be detected. (**B**) The line annotated with “1” in the side position indicates the starting position of the scanning at which the greater (outer) curvature of the stomach and cross-section of the spleen is detected, whereas “2” indicates the approximate position at which a cross-section of the spleen tumor and kidney can be visualized.

**Figure 2:**
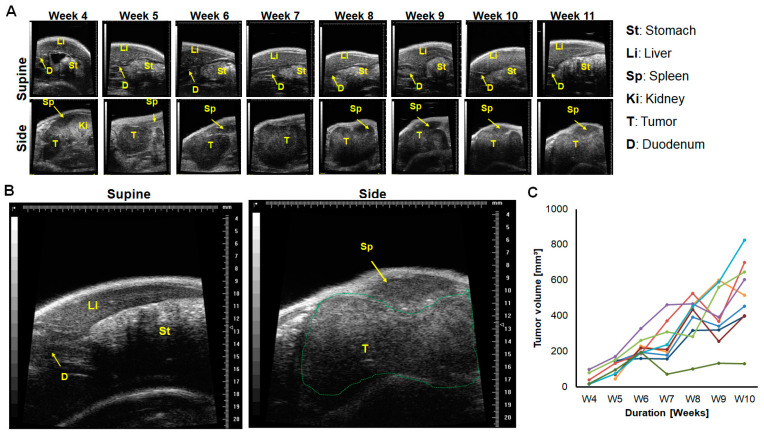
Representative ultrasound images of a mouse with an orthotopic tumor secluded within the pancreas. (**A**) Images acquired once weekly from week 4 to week 11 post tumor implantation. Imaging in the supine position ensures visualization of the liver, stomach, and duodenum, while imaging from the side position reveals the secluded tumor within the pancreas and also the neighboring spleen and kidney. (**B**) Enlarged images of the mouse at week 11 showing the liver, stomach, and duodenum (supine) and the spleen and large tumor (green broken line) that partially outgrew the pancreas and outsized the field of view of the ultrasound system used (side). Of every 10 mice bearing Panc-1-fl tumors, n = 4 did not infiltrate the omentum. (**C**) Tumor growth curves of individual mice from week (wk) 4 to 10 post induction. Each colored line depicts tumor growth of a single mouse.

To be able to make accurate estimations on the progression of implanted orthotopic pancreatic tumors in mice, each mouse was imaged by ultrasound both in the supine and side positions (Figure 1). In the supine position, the visualization of the liver with blood vessels, the stomach, and duodenum permitted the detection of their infiltration by tumor cells. In about 40% of mice implanted with Panc-1-fl cells in this study, the tumors grew and remained secluded within the pancreas. Thus, we could observe a clear liver, stomach, and duodenum without evident infiltration from 4 weeks up to 11 weeks post tumor implantation (Figure 2A,B, supine). Imaging the same mice in the side position revealed the greater curvature of the stomach, the spleen, and the pancreas with the secluded primary tumor (Figure 2A,B, side). As the growth of the secluded primary tumor progressed over time, its ultrasound-based visualization became more evident. Interestingly, in 40% of the mice investigated, the secluded tumors almost outgrew the pancreas from week 9 post induction (Supplementary Video S1) and outsized the widest field of view (FOV) of the Vevo700 ultrasound system used (see Figure 2B, side). This influenced the accuracy of the tumor volumes determined at advanced tumor growth stages. The deduced tumor volumes using the ultrasound system fluctuated and reached an average of 600 mm^3^ in yjr later weeks of growth (Figure 2C).

### 2.2. Tumor Infiltration of Vital Organs and Disease Progression

**Figure 3:**
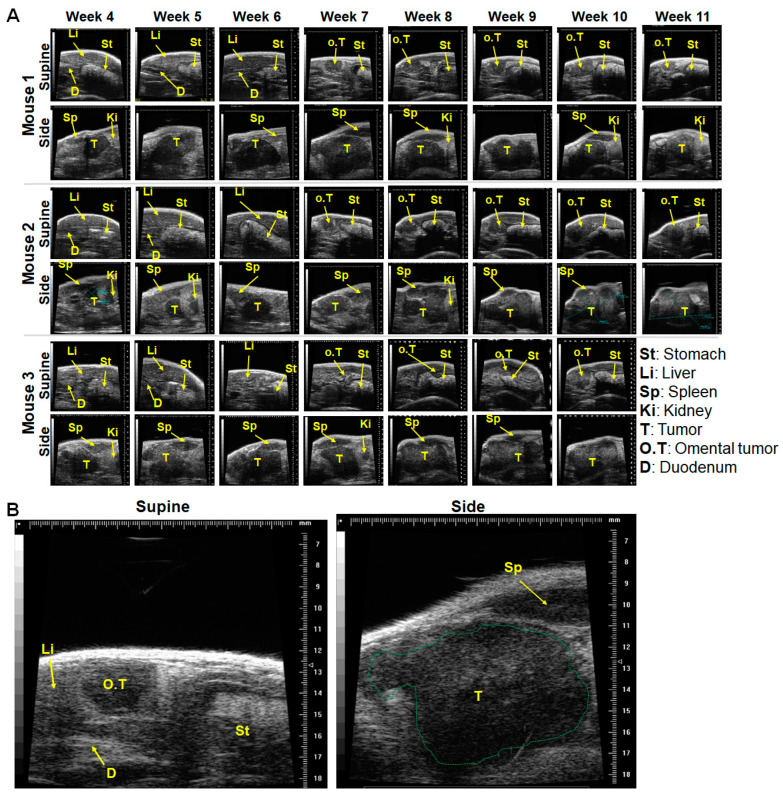
Ultrasound images of mice with orthotopic Panc-1-fl tumors in the pancreas and detectable infiltration of the omentum. (**A**) Images acquired once weekly from week 4 to week 10 or 11 post tumor implantation. Imaging in the supine position enables visualization of the liver (Li), stomach (St), and duodenum (D) and an omental tumor (O.T.) seen lying between the stomach and duodenum as from week 7 onwards. Imaging from the side position detects the primary orthotopic tumor (T) within the pancreas, and also the neighboring spleen (Sp) and kidney (Ki). (**B**) Enlarged images of the “Mouse 1” at week 7 showing part of the liver, and the link between stomach and duodenum being partially occupied by the omental tumor (supine); and the spleen and large primary orthotopic tumor (green broken-line) within the pancreas and showing the emanating growth towards omentum (side). Of every 10 mice bearing Panc-1-fl tumors, n = 6 infiltrated the omentum.

As can be deduced from the images (Figure 3A,B, supine), we observed the tumor outgrowth and infiltration of the omentum. This was detected in 60% of the investigated Panc-1-fl tumor bearing mice. Omental infiltration was detected in the supine position of mice, as small dark tumor lying close to/on the stomach as from week 7 post Panc-1-fl tumor implantation (Figure 3A, supine). In contrast, omental infiltration by the BxPC-3 tumor model occurred earlier (at week 5) and was less peculiar than the massive infiltration of the stomach wall by the BxPC-3 model (Supplementary Figure S2 and Supplementary Video S3). It is noteworthy that the blood parameters of mice bearing the BxPC-3 and the Panc-1-fl tumor models were almost identical (Supplementary Figure S3), indicating that the overall burden of the tumors on mice blood parameters, especially platelets that protect circulating tumor cells was not influenced in the BxPC-3 tumor bearing mice.

The omentum in the mouse is a small, elongated fat tissue that extends from the pancreas splenic lobe to the stomach area near the pancreas gastric lobe (see Supplementary Figure S1 and [33]). The native omentum was not detectable in ultrasound imaging, except when infiltrated, for example, by tumor cells. The Panc-1-fl omental tumors were initially visualized close to the pyloric sphincter between the stomach and the duodenum at the onset of infiltration (Figure 3B, supine) and as an elongated tumor in the later stages of growth (Supplementary Video S2B,C). When imaging the same mice on their side positions, the spleen and the pancreas with the primary tumors were clearly distinguishable, and their progression monitored over time, independent of the omental tumors. This suggested that the omental infiltration probably stems from the gastric lobe of the mice pancreas, which based on our observations (see Supplementary Figure S1), is located close to the intersection between the stomach and duodenum. This observation reiterates the importance of additionally monitoring orthotopic human pancreatic tumor bearing mice in the supine position. It also indicates that it is vital to consider the omental tumor when estimating the overall tumor mass in a given mouse over time.

### 2.3. Validation of the In Situ Positions of Tumors and Organs by Near-Infrared (NIR) Fluorescence Imaging

**Figure 4:**
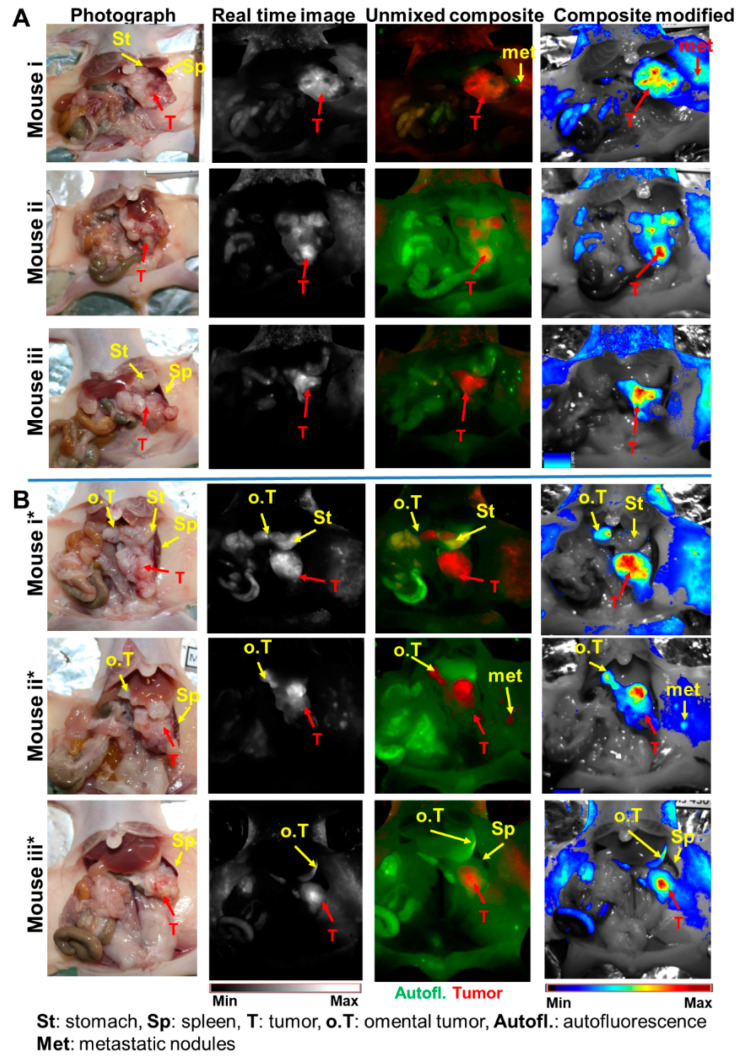
Near-infrared fluorescence imaging validated the positions of orthotopic tumors within the pancreas, infiltrated omentum, and metastatic nodules. (**A**) Images of the situs of dissected mice that revealed a secluded Panc-1-fl primary tumor within the pancreas in ultrasound imaging. The photographs show the large tumors within the pancreas, whereas the fluorescence images substantiate the tumor location and mild dissemination to the muscle/skin that was close to the tumor (denoted met in Mouse i). (**B**) Photograph and corresponding fluorescence images of the situs of dissected mice that revealed orthotopic primary tumors within the pancreas and detectable omental infiltration in ultrasound imaging. The orthotopic primary tumors within the pancreas (T) as well as the small, elongated omental tumors (o.T) close to the stomach (St) can be seen in the photographs and validated by their fluorescence signals. Also, a few metastatic nodules (met) are seen (Mouse ii*).

During the dissection of mice at weeks 10–11 post tumor induction (n = 10), the near-infrared fluorescence imaging of the situs pinpointed the tumor and infiltration of neighboring organs. This was enabled by the mkate2 protein fluorescence of the implanted Panc-1-fl cells. Mice that revealed secluded tumors within the pancreas during ultrasound imaging also showed the fluorescence of the large secluded tumor (Figure 4A). However, a few fluorescent dots were evident on the muscle/skin close to the tumor in some mice (Figure 4, Mouse i and ii*, met). Interestingly, except for a few tiny metastatic nodules that were missed by the ultrasound, the fluorescence signals of the orthotopic primary tumors and omental infiltrates correlated well with observations made by ultrasound imaging. This suggests that ultrasound imaging combined with optical imaging could be useful for grading the growth advancement of pancreatic cancer models for preclinical drug testing purposes (Figure 4B, Mouse ii*).

**Figure 5:**
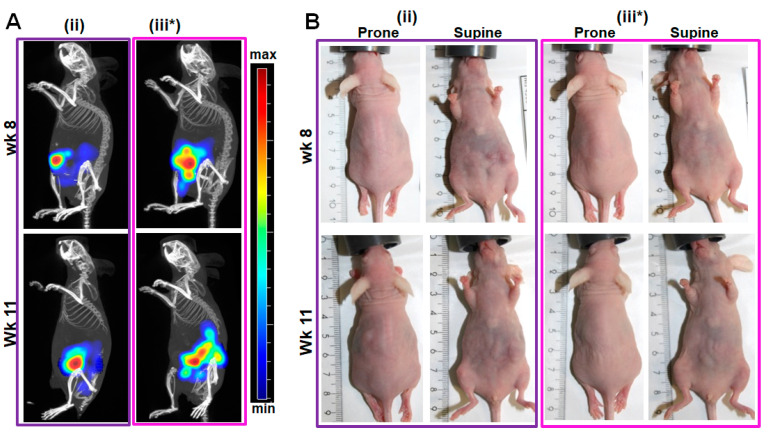
Fluorescence imaging tomography localizes tumors deeper within the mouse tissue. (**A**) Fluorescence tomographic images of a mouse with no omental infiltration (denoted (ii) and corresponding to Mouse ii of Figure 4A) and one with omental infiltration (denoted (iii*) and corresponding to Mouse iii* of Figure 4B). (**B**) Photographs of the mice at week 8 and 11 prior to tomographic imaging reveals protrusion of the tumor in (ii) especially in the supine position, which does not directly correlate with the detected fluorescence signals.

Fluorescence imaging tomography allows the detection of tumors lying deeper in the tissue. For example, the tumor fluorescence at week 8 and week 11 post induction was detected as somewhat diffuse signals of the mkate2 fluorescence in the implanted Panc-1-fl cells. Only minimal differences in intensity were noted between the time points. In this setup detecting fluorescent tumor cells and not an injected contrast agent, tomographic fluorescence imaging was not suitable for the estimation of tumor sizes or metastasis locations (Figure 5). The fluorescence signals did not enable a clear demarcation of the omental infiltration (Figure 5A), nor did they correlate with the tumor sizes when considering the palpable or visible tumors in situs at week 11 (compare Figure 5 signals with tumors in Figure 4, Mouse ii and Mouse iii*). 

### 2.4. Comparative Validation of the Mouse Pancreas Anatomy with Surrounding Organs, and Excised Tumors

**Figure 6:**
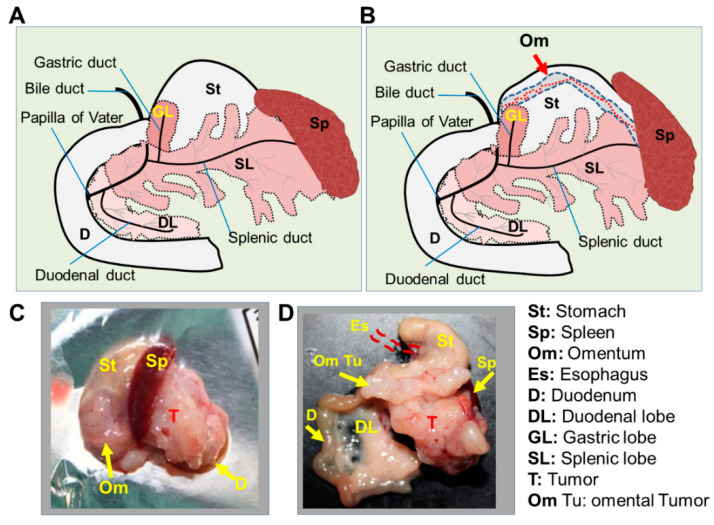
Validation of the anatomical locations of implanted tumors. Schematic drawing of the macroscopic anatomy of mouse pancreas and surrounding organs according to Dolenšek et al. [25] omitting the omental fat (**A**,**B**) with consideration of the omental fat according to personal observations (Supplementary Figure S1) and the authors of [33]. The mouse pancreas comprises the splenic, gastric, and duodenal lobes. (**C**) Representative photograph of excised tumors with surrounding organs without visible tumor infiltration of the omentum. The very large primary tumor filled up the splenic and duodenal lobes and almost outgrew the pancreas. (**D**) Representative photograph of tumor and surrounding organs that were excised from a mouse with omental infiltration according to ultrasound imaging. The large primary tumor did not grow towards the duodenal lobe (DL) but rather towards the omentum, visible as an elongated omental tumor (om Tu). Of every 10 mice bearing Panc-1-fl tumors, n = 6 showed omental infiltration.

To understand the images of orthotopic pancreatic tumors and location relative to the surrounding organs accurately, we verified some details on the mouse pancreas anatomy. The mouse pancreas comprises the splenic lobe (SL), gastric lobe (GL), and duodenal lobe (DL). A closer look at the mouse pancreas anatomy reveals similarities to human pancreas [25] (Figure 6A). The omentum in mice is a very thin visceral fatty tissue (see Supplementary Figure S1 and reference [33]) that is located across the greater (outer) curvature of the stomach. It is linked to the pancreas splenic lobe (SL) and extends to the pancreas gastric lobe (GL) at the intersection of the stomach and duodenum (Figure 6B, see also Supplementary Figure S1 and [33]). Interestingly, the excision of tumors together with surrounding organs revealed a free non-infiltrated omentum in some mice (Figure 6C) and some fully infiltrated omentum (Figure 6C,D). It is likely that the growth of the tumor towards the omentum is triggered by activating signals from the omentum.

### 2.5. Immunohistochemistry Revealed Differences in the Levels of Naïve Pancreatic Stellate Cells and Mature Fibroblasts in the Primary and Omental Tumors over Growth Time

**Figure 7:**
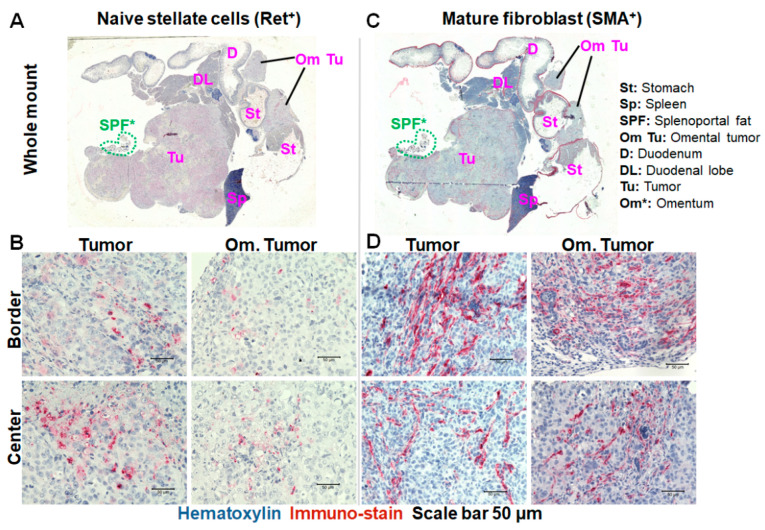
Representative light microscopic images of histological slices of a Panc-1-fl orthotopic tumor and surrounding organs with visible infiltration of the omental fat as seen in ultrasound imaging at week 10 after implantation. (**A**) Whole section mount at 4× and (**B**) extracts of the tumor at a 40× magnification after staining vitamin A in naïve pancreatic stellate cells. (**C**) Whole section mount at 4× and (**D**) extracts of the tumor at a 40× magnification after staining the alpha-smooth muscle actin (alpha-SMA) in mature tumor associated fibroblasts (myCAFs). The primary tumor can be seen within the pancreas, whereas the omental tumor (om Tu) is seen lying at the intersect between the duodenum and stomach and extending along the wall of the stomach. SPF* (circled in green broken lines in (**A**,**C**)) indicates a tumor-free section of the splenoportal fat.

**Figure 8:**
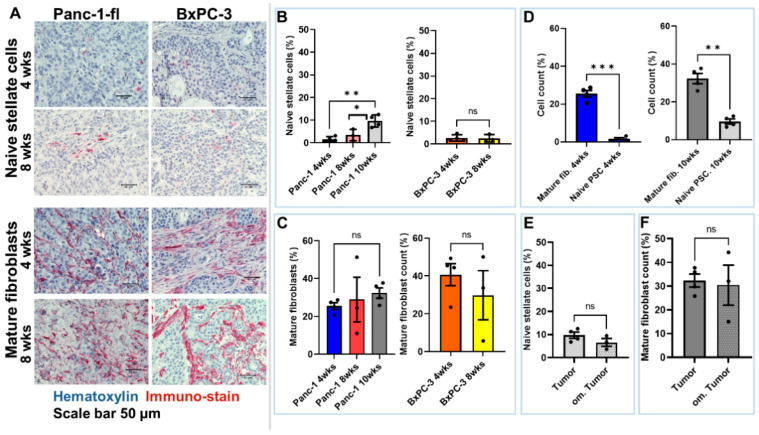
Semiquantitative analysis of naïve pancreatic stellate cells and mature fibroblasts in slices of Panc-1-fl and BxPC-3 tumors excised at 4 weeks, 8 weeks, and 10 weeks post implantation. Microscopic images of stained tumor regions were acquired at 40× magnification. (**A**) Representative light microscopic images of 4- and 8-week-old Panc-1-fl and BxPC-3 tumors showing the naïve pancreatic stellate cells (PSCs) and mature fibroblasts. (**B**–**F**) The level of naïve PSCs (based on vitamin A level) and mature fibroblasts (based on alpha-SMA expression) were deduced from the images by cell counts using ImageJ software version 1.8. Each bar represents the relative number of stained cells within the slices of 1–3 animals. Each dot represents the mean of 4 images per mouse tissue, except for week 4 where the dots represent different slices from the same mouse. PSC: pancreatic stellate cells, wks: weeks, fib.: fibroblast. Error bars represent standard deviations (S.D.), ns.: non significant. * *p* < 0.05, ** *p* < 0.006, *** *p* < 0.0005 as determined by unpaired *t* test with Welch correction. (**B**) Level of naïve stellate cells in 4- to 10-week-old Panc-1-fl and 4- to 8-week-old BxPC-3 tumors. (**C**) Level of mature fibroblasts in 4- to 10-week-old Panc-1-fl and 4- to 8-week-old BxPC-3 tumors. (**D**) Comparison of mature fibroblasts versus naïve stellate cells in 4-week-old and 10-week-old Panc-1-fl tumors. Comparison of naïve stellate cells (**E**) and mature fibroblasts (**F**) in Panc-1-fl primary versus omental tumors.

Tumor slices of some mice, specifically 1 mouse for week 4, 2 mice for week 8 and 3–6 mice for week 10/11 tumors were evaluated. A comparison of naïve vitamin A-positive pancreatic stellate cells (naïve PSCs) and mature smooth muscle actin-positive fibroblasts (myCAFs) in the tumors validated their progression over time (Figure 7 and Figure 8). Generally, a lower number of vitamin A-positive naïve stellate cells was seen in tumors at all stages, compared to mature fibroblasts (Figure 7A,B and Figure 8A). These were partly in the process of transformation towards mature fibroblasts as seen in their elongated morphology. This was opposed to the somewhat rounded morphology of naïve stellate cells with strong intensities of Vitamin A deposits observed in week 4 and week 8 tumors (Figure 7B and Figure 8A). Furthermore, the strong fibrosis of the tumors, evident in the abundance of smooth muscle actin positivity and elongated morphology of the mature fibroblasts was seen in the tumors (Figure 7C,D and Figure 8A). This was evident in both the primary tumor and omental tumor infiltrates (Figure 7A–D). The observations validate the omental tumors seen in ultrasound imaging. Additionally, a tumor-free section of the splenoportal fat (SPF, see Supplementary Figure S1A “situs back view”) could be detected (Figure 7A,C; green broken lined circles). As seen in the images, the SPF connects directly to the splenic lobe of the pancreas (now with a large tumor) at a position opposed to the side where the omental fat connects to the splenic lobe. The fact that this SPF was not infiltrated by tumor cells as was the case with the omentum suggests their lack of factors that attract the tumor cells.

The semi-quantitative assessment of the number of vitamin A containing naïve stellate cells revealed a continuous increase over time, with a significantly higher number of stellate cells in 10 weeks than in 4-week-old (** *p* < 0.006) and 8-week-old (* *p* < 0.05) Panc-1-fl tumors (Figure 8B). This suggests a slow infiltration of the tumors by naïve stellate cells. In contrast, the level of mature fibroblasts in the Panc-1-fl tumors increased over time but was not statistically different between week 4, 8, and 10 tumors (Figure 8C), suggesting that the infiltration of the tumors by fibroblasts is quite rapid and reaches a maximum before week 8. Furthermore, it is likely that the further increase in mature fibroblasts from week 8 onwards results from the gradual transformation of naïve vitamin A-containing stellate cells in the tumors. In line with this, the aggressive BxPC-3 tumors which attained a late metastatic stage earlier after induction (see Supplementary Figure S2) revealed no difference in the levels of naïve stellate cells between week 4 and week 8 tumors (Figure 8B). Furthermore, very high levels of mature fibroblasts were detected, with no difference between 4-week-old and 8-week-old BxPC-3 tumors (Figure 8C).

Interestingly, both tumor models revealed significantly higher levels of mature fibroblasts as compared to naïve stellate cells at all the investigated time points, as shown, for example, in 4-week-old (*** *p* < 0.0005) and 10-week-old (** *p* < 0.006) Panc-1-fl tumors (Figure 8D). This was also the case when comparing the level of mature fibroblasts with naïve stellate cells within omental Panc-1-fl tumors at 10 weeks after induction. However, a comparison of naïve stellate cells or mature fibroblasts in primary tumors versus omental tumors revealed no significant difference (Figure 8E,F). In the quantification of naïve vitamin A-containing stellate cells, we did not differentiate cells based on morphology. It must be noted, that more rounded cells and less elongated cells were visible in week 4 as compared to week 8 and older tumors (Figure 7B and Figure 8A). This substantiates an increase in the maturation of the tumors over time post implantation.

### 2.6. Collagen Fiber Levels Support Progression-Related Differences in the Tumors

**Figure 9:**
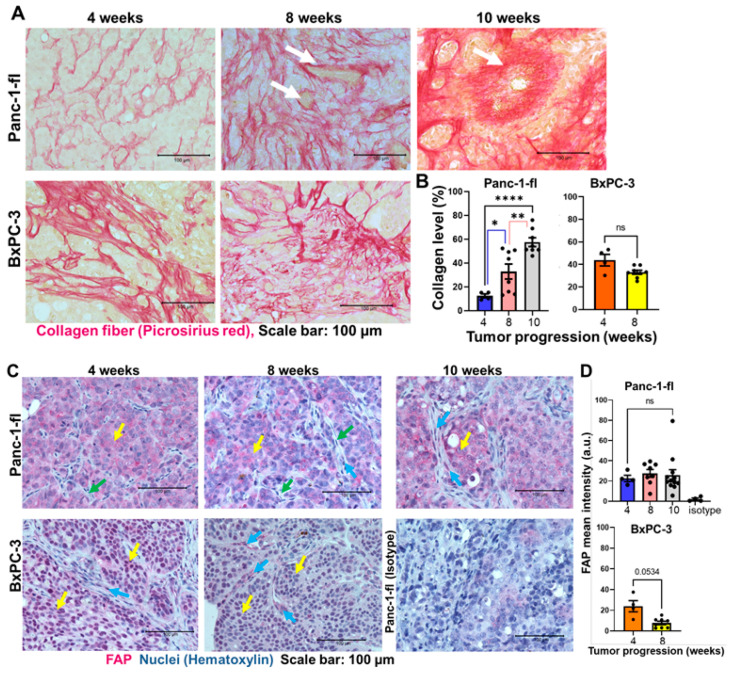
Analysis of collagen fiber and FAP levels in orthotopic pancreatic tumors during growth. Representative light microscopic images of histological slices of (**A**) Panc-1-fl and BxPC-3 tumors acquired at 40× magnification after staining collagen fibers with picrosirius red reagent. The level of collagen fibers is evident in the intense red stain, also seen surrounding blood vessels (white arrows). (**B**) Semi-quantitative evaluation of the level of collagen fibers in microscopic images. There was an increase in collagen over the observed growth time in Panc-1-fl tumors and stagnation in the BxPC-3 tumors after 4 weeks. Each bar represents the relative area of tumor with collagen stain within the slices of 1–2 animals and four images per tumor slice. Error bars represent standard error of means (SEM.). * *p* < 0.02, ** *p* < 0.006, **** *p* < 0.0001 as determined by unpaired *t*-test with Welch correction. (**C**) Representative light microscopic images of Panc-1-fl and BxPC-3 tumor slices acquired at 40× magnification after immunostaining FAP. The tumor cells express FAP (yellow arrows). In contrast, most cells within the stroma of the week 4 and 8 Panc-1-fl tumors show no FAP stain (green arrows). Only very few elongated stroma cells are positive for FAP in the 8-week Panc-1-fl tumors (blue arrows). The 10-week-old Panc-1-fl tumors as well as the 4- and 8-week-old BxPC-3 models show increased stromal FAP signals (blue arrows). (**D**) Semi-quantitative evaluation of FAP mean intensities (of all cells) show no significant differences in FAP levels over the growth period. BxPC-3 shows a slight tendency of decrease of overall FAP (*p* = 0.0534 (n.s.) for week 4 versus week 8 tumors).

The histological slices of tumors excised at weeks 4, 8, and 10 were stained with picrosirius red for collagen fiber detection as described in the Section 4. Light microscopy images acquired at 40x magnification revealed smaller collagen fibers in week 4 Panc-1-fl tumors, and an increase in the collagen bundle size in 8 weeks and 10 weeks tumors (Figure 9A, Panc-1-fl). These were peculiar as bundles surrounding blood vessels in week 8 and 10 tumors (Figure 9A, white arrows). In contrast, the BxPC-3 tumors showed large collagen bundles already at 4 weeks after induction (Figure 9A, BxPC-3), indicating its rapid and aggressive progression.

The semi-quantitative analysis of the area of tumors covered by collagen substantiated a continuous increase in collagen levels over time in the Panc-1-fl tumors (Figure 9B, Panc-1-fl). Thus, a significantly higher level of collagen was seen in the 8-week-old (* *p* < 0.02) or 10-week-old Panc-1-fl tumors (**** *p* < 0.0001) than the week 4 tumors. Moreover, more collagen was detected in 10-week-old than in 8-week-old Panc-1-fl tumors (** *p* < 0.006). Interestingly, the level of collagen as compared to alpha-SMA expressing cancer fibroblasts (myCAFs) was significantly lower in the 4-week Panc-1-fl tumor but increased to same level as myCAFs in 8-week tumors, and became significantly higher (*** *p* < 0.0005) than myCAFs in the 10-week tumors (Supplementary Figure S4). This signifies a persistent progression of the tumors towards late and highly desmoplastic stages. In contrast, the BxPC-3 tumors excised at 4 and 8 weeks after induction showed no significant differences in collagen levels (Figure 9B, BxPC-3), suggesting that the tumors reached an advanced stage and a collagen plateau by week 4 after implantation, indicating their aggressiveness.

Compared to the collagen levels that increased significantly as tumor growth progressed, FAP levels remained unchanged during progression. Interestingly, the pancreatic cancer cells express endogenous FAP [34]. This made it difficult to distinguish between the murine FAP-expressing inflammatory cancer-associated fibroblasts (iCAFs) from the human FAP expressing tumor cells (Figure 9C,D), owing to the known antibody cross-reactivity for human and mouse FAP [35]. However, the images revealed the predominant stain of the tumor cells (Figure 9C, yellow arrows) and clearly unstained elongated stromal cells for the 4- and 8-week-old Panc-1-fl tumors (Figure 9C, green arrows) with few stromal cells stained in the 8-week tumor. In contrast, the 10-week-old Panc-1-fl model revealed FAP stain of the tumor cells and also the elongated stromal cells (Figure 9C, blue arrows). Likewise, and quite interestingly, for the BxPC-3 model, FAP was expressed in the tumor cells (Figure 9C, yellow arrows) and the stromal cells in 4- and 8-week-old tumors (Figure 9C, blue arrows). Therefore, the intensity of the FAP signals of stromal cells became higher than that of tumor cells in the 8-week-old BxPC-3 tumors. Remarkably, while the FAP in Panc-1-fl tumors was diffusely distributed in the tumor cells, these were more nuclear localized in the BxPC-3 models. A relative count of the FAP-positive tiny elongated stromal cells revealed low levels in week 4 tumors and an increase in both tumors from week 8 onwards (Supplementary Figure S6). Taken together, these observations suggest that the maturity of the tumors is closely linked to the expression of FAP in stromal cells.

## 3. Discussion

PDACs cause devastating problems to patients. Therefore, continuous efforts in the design of new treatment options and their initial preclinical testing are inevitable for their future management. The identification of suitable murine orthotopic tumor models and the features that define their progression towards advanced and late disease stages can guide their inclusion in preclinical drug testing procedures and thereby improve the selection of therapeutics and treatment regimens customized for the effective elimination of different progression-dependent parameter-based stages of diseases. In this investigation, we established a reproducible human cell-based orthotopic pancreatic tumor model in mice and validated some features that characterize their development. The identified features (morphological imaging features and levels of PSCs, myCAFs, iCAFs, and collagen) allow the estimation of different tumor growth stages that can be considered as a starting point for the analysis of therapeutic effects of drugs on tumor development.

Morphologically, early-stage primary tumors were secluded within the pancreas, with no metastatic infiltrate in organs or omental fat detected by ultrasound imaging. The advanced and late stages almost outgrew the pancreas, infiltrated the omental tissue, spread as metastases to neighboring organs, and infiltrated the stomach wall (for BxPC-3). An accurate imaging of the morphological features of orthotopic pancreatic tumor advancement requires imaging from both the side and supine positions. For example, the infiltration of the stomach or omental tissue at approximately 5 weeks post implantation of BxPC-3 tumors and approximately 7 weeks post implantation of the Panc-1-fl cells respectively were detected by ultrasound imaging in the supine position. The omentum in mice is a small, elongated visceral adipose tissue lying slightly ventral to the stomach [33,36]. The omentum secretes many pro-tumorigenic factors and exhibits known immune functions in mice and humans [33,37,38,39]. Opposed to other peritoneal adipose tissues such as gonadal or uterine fat, it is a colonization site for metastatic cancer cells [40]. Both preclinical and clinical findings show that omental carcinomatosis is common in pancreatic (e.g., clinical [41]), gastric, ovarian, and colorectal cancers and is associated with the late stage of the disease, chemotherapy resistance, and poor prognosis [40,42,43,44,45,46]. Hence, the identification of the onset of omental infiltration exposes disease advancement in mice tumor models.

At the molecular level, the BxPC-3 model showed high numbers of αSMA-positive myofibroblasts (myCAFs) and collagen fibers and low levels of vitamin A-containing naïve pancreatic stellate cells (PSCs). It was demonstrated that pancreatic cancers have two characteristic fibroblasts subpopulations, namely myofibroblastic ones based on the expression of αSMA (myCAFs) and an inflammatory subset (iCAFs) based on fibroblast activation protein (FAP) expression, amongst other markers [32]. Hence, increased levels of activated fibroblasts and collagen in tumors are features of advanced and late disease stages. These appeared already at 4–8 weeks post implantation of the BxPC-3 cells (see Supplementary Figures S2 and S5, and Figure 8 and Figure 9), substantiating a more aggressive nature. Research reports showed that the *KRAS*-mutant Panc-1 cells migrate as single cells, whereas the *KRAS* wild-type BxPC-3 migrated as a complete monolayer sheet in cell migration assays [47]. This could contribute to the sheet-like infiltration of the stomach wall, though pancreatic adenocarcinoma metastasis of the stomach is rarely reported in humans [48]. However, this may be partly because the metastases were missed. Interestingly, pancreatic cancer metastasis of the stomach has been detected in autopsy cases [49] and raised awareness for its possible occurrence and hence vigilance during clinical diagnosis. One research work in which stomach metastasis was detected revealed that metastatic spread was irrespective of *KRAS* status but rather depended on the number of passages in culture [50]. Though we cannot eliminate this possibility in our BxPC-3 model, our observations indicate that the BxPC-3 is an example of the orthotopic pancreatic tumor model with rapid growth and early advancement. This is likely due to the beneficial pancreas environment of the orthotopic setup and suggests that starting imaging as early as 1–2 weeks post implantation and following up the development continuously are vital.

Contrary to BxPC-3, the Panc-1-fl model revealed a slower growth progression, a higher rate (60%) of omental infiltration and no stomach wall infiltration. At the molecular level, the Panc-1-fl tumor model revealed high levels of αSMA-positive mature fibroblasts, low vitamin A-containing PSCs and high collagen bundles over the growth time, exposing their progression towards late fibrotic stages. According to the literature, the levels of the naïve PSCs decline in tumors with advanced progression. However, in the orthotopic model presented herein, where only human tumor cells were implanted, the entire stroma, including the quiescent pancreatic stellate cells and mature fibroblasts, originate from the host. Hence, it was not surprising to determine an increase in vitamin A-containing PSCs in the Panc-1-fl tumors from 4 weeks to 10 weeks after tumor induction but no further increase for the BxPC-3 model as from 4 weeks after implantation. Naive or quiescent pancreatic stellate cells are rich in vitamin A (retinol) and play significant roles in remodeling the pancreatic extracellular matrix. Their activation by different factors, including, for example, IL-1, IL-6, TNF-α, TGF-β1, and activin 1 [51,52], induces fibrosis and accounts for the desmoplastic property of pancreatic adenocarcinomas. At the onset of carcinomatosis in the pancreas, these growth factors are secreted by macrophages and pancreatic acinar cells. As the tumor growth advances, the tumor cells themselves produce growth factors, for example, PDGF, FGF-2, and TGF-β, which activate stellate cells and their production of ECM proteins [53]. Once activated, the mature fibroblasts (myCAFs and iCAFs) further enhance the transformation of vitamin A-containing naïve PSCs into mature fibroblasts via the secretion of TGF-β1 and activin [51]. This aggravates collagen accumulation and desmoplasia in pancreatic adenocarcinomas.

The relatively low level of naïve pancreatic stellate cells detected in both orthotopic tumor models, and their elongated morphology indicative of their transformation into mature fibroblasts, further substantiates the progression of the tumors through different stages. Furthermore, the presence of FAP-positive elongated stromal cells (iCAFs) in both week 4 and 8 BxPC-3 tumors, but predominantly in week 8 and 10 and negligible in week 4 Panc-1-fl tumors (Supplementary Figure S6), indicates that FAP-expressing iCAFs mark an advanced tumor stage in the mouse models. Thus, it is conceivable that the detection of omental infiltration and other morphological features of orthotopic PDAC models and their alignment with molecular features for the identification of “growth stages” is feasible. The purposeful testing of therapeutic options on such defined “tumor growth stages” would improve the selection of efficient therapeutic drugs and strategies to combat pancreatic cancers.

Ultrasound imaging is non-invasive and relatively cost-effective and provides anatomical information of soft tissues. Nevertheless, its low penetration depth is limiting, especially using the classic Vevo700 ultrasound system as we did. With the evolution of advanced ultrasound systems that provide contrast-agent-enhanced molecular and functional information of the tumors, the accuracy of its single use for defining tumor progression-based features could be improved. Molecular imaging methods like fluorescence and bioluminescence are also relatively cost-effective and can additionally provide vital information. Nonetheless, they are limited by the poor penetration of light. Our use of fluorescence imaging tomography (FLIT) based on the fluorescent mkate2 protein was not suitable for the estimation of tumor sizes, whereas the planar fluorescence imaging of the situs detected tiny metastases. Additionally, the diffuse FLIT signals could not correctly distinguish between primary tumors and infiltrated neighboring organs in live animals (see Figure 5). For an accurate estimation of tumor growth by FLIT, it would be necessary to use an additional tumor-targeting fluorescent agent. Furthermore, additional histological analysis (e.g., of biopsies) and consideration of functional and molecular imaging or methods with high penetration depth, for example, magnetic resonance imaging (MRI), positron emission tomography (PET), amongst others [54], would considerably improve the accuracy of the “preclinical progression parameter-based tumor staging” proposed herewith.

### Summary and Suggestions for the Designing, Monitoring, and Selection of Cell- or Graft-Based Orthothopic Pancreatic Tumor Models Based on Growth-Dependent Tumor Parameters Prior to Preclinical Drug Testing

The underlying study points out tumor-progression-dependent parameters of human cell-based orthotopic pancreatic tumor models in mice, which can help standardize preclinical research (Table 1). We believe that a tumor-growth-parameter-oriented selection of tumors for preclinical therapy testing can improve the future identification of drugs tailored for tumor growth stages characterized by defined progression-dependent signatures.

It must be noted that cancers are highly heterogenic, and the features described here with the two PDAC cell lines are not unique for all PDAC models. The implementation of other cell lines with different properties would prospectively lead to the detection of further features that can be considered to group preclinical tumor models for drug testing. Considering this, together with our observations (Table 1), we suggest the following steps for the planning and preclinical testing of therapeutics and new treatment options for pancreatic cancer models in mice in the future.

i For the preparation of cells and tumor fragments for implantation, a low concentration (2–4% (*v*/*v*) depending on the tumor model in question) of a supporting matrix should be added before implantation, and the implantation should be performed directly into the pancreas body (splenic lobe).
ii Ultrasound imaging should be carried out preferentially both on the side and supine position in order to monitor the primary tumor and to pinpoint the onset of the infiltration of the omentum and/or stomach wall and metastasis. Furthermore, the use of imaging systems that have higher penetration and/or additionally provide functional and molecular information of the tumors [54] should be considered.
iii For therapy efficacy testing purposes, a designated “tumor growth stage” based on the progression-dependent characteristics of the model in question (see Table 1 above) should be selected in order to define suitable therapeutic drugs efficient for the parameter based early, advanced, or late stages.

We admit that monitoring tumor growth from week 4 after tumor implantation as we did (based on the cells’ long doubling time of approximately 56 h) might have missed critical molecular features relevant for the correct staging of the tumors. To consider this and thereby circumvent the excessive use of mice, biopsies of the tumors should be considered for molecular assessments in the future. Also, although the growth of PDAC does not require sex-related hormones, hence our use of only female immune-deficient athymic nude mice in this study, the inclusion of male mice and methods that allow tumor growth in immune competent mice should also be considered in future studies. Additionally, using more modern ultrasound systems that are equipped with modules for detailed analysis of tumor blood flow, elasticity, and other specifications would boost the estimation of tumor progressional stages. Notwithstanding, we are convinced that the presented data are insightful suggestions to researchers aiming to design and test therapeutics and methodologies for the management of pancreatic and other cancers. Furthermore, it should serve as food for thought when planning future research studies.

## 4. Materials and Methods

### 4.1. Cell lines and Cell Culture

Different pancreatic cancer cell lines have different growth, metastasis, and drug response potentials. We used the gemcitabine-resistant [26] human PDAC cell lines Panc-1 [27] and BxPC-3 [28]. BxPC-3 contains a wild-type *KRAS* oncogene, and Panc-1 has a mutant *KRAS*, hence their inclusion in this study. The Panc-1 cells were made fluorescent by the stable expression of mkate2, a super bright, far-red fluorescent derivative of the katushka protein [55]. This was important since the subsequent fluorescence tracking of metastasizing cells and circulating tumor cells was planned. In our experience, no differences exist in the cell and tumor growth between the mkate2-based fluorescent Panc-1 (termed Panc-1-fl) and wild-type Panc-1 cells. The cells were cultured in an RPMI-1640 medium (Gibco^TM^ 21875034, code 11530586), under standard culture conditions with 5% CO_2_ and 95% humidity. For sub-culturing and preparation for implantation, the cells were washed 3 times with Hanks’ Balanced Salt Solution (HBSS, Cellgro, Kaiserslautern, Germany, cat. no. 21-021-CV) trypsinized with 0.5% (*w*/*v*) trypsin–EDTA (Gibco, /ThermoScientific, Schwerte, Germany, cat. no. 15400), then dispensed in medium containing serum, counted, and centrifuged. The final cell pellet was dispensed either in growth medium for further culturing or in HBSS-based implantation solution (HBSS-Matrigel^®^) as detailed below.

### 4.2. Use of Animals

All procedures involving animals were performed in accordance with institutional regulations and in conformation with international guidelines on the ethical use of animals. This was approved by the regional ethical committee in charge of animal use and care (Thüringer Landesamt für Verbraucherschutz, Bad Langensalza, Germany), under the registry number UKJ-17-030. All animals were housed under standard conditions (14 h/10 h light–dark cycles; 25 °C temperature) with food and water supply ad libitum.

#### Immune Deficient Mice

Common immune deficient mice strains, for example, athymic nude mice, SCID, or NOD/SCID mice, are well suited for the propagation of orthotopic pancreatic tumor models. Our experience with heterotopic cancer models reveals that the athymic nude and the SCID mice strains show no difference in the tumor formation capacity. We hence used 30 female nude mice (Rj:Athym-Foxn1^nu/nu^, Janvier, Le Genest-Saint-Isle, France) in this study due to the ease of handling and the intended ultrasound imaging of the tumor progression over a long duration. These mice were 6–8 weeks old at the time of tumor implantation.

### 4.3. Generation of Pancreatic Cancer Models in Mice

Reliable protocols published by other groups describe the heterotopic and orthotopic implantation of cancer cell lines or engraftments of human primary pancreatic tumors in mice [15,16]. Since different cell lines have different growth and metastasis potentials, the protocol by Kim and colleagues [16] was used in this study with minor modifications to generate the orthotopic tumor models of the human PDAC cell lines Panc-1 and BxPC-3 in mice. Briefly, cell suspensions were prepared in 2% (*v*/*v*) Matrigel^®^ Growth Factor Reduced, Basement Membrane Matrix (Corning^®^, Corning, NY, USA, cat. no. 356230) solution in sterile HBSS. The suspension was drawn into a sterile 1 mL tuberculin syringe, using a sterile 27G needle (Sterican^®^, B.Braun Melsungen AG, Melsungen, Germany, PZN: 02499682). The syringe with the cell suspension was placed at 4 °C on ice for implantation. The total volume for each cell suspension was 100 µL and contained 4 × 10^6^ cells or 2 × 10^6^ cells for Panc-1-fl and BxPC-3, respectively. A final volume of 75 µL was injected into the pancreas body (splenic lobe). Ten mice were implanted with BxPC-3 cells and twenty mice with Panc-1-fl cells. Thereby, it was considered that the doubling times of the cells is approximately 56 h and the tumor growth success after surgical procedure was 80–90%.

#### Microsurgical Procedure for Tumor Cell Implantation into the Pancreas and Expected Duration for Tumor Development

Three days prior to tumor cell implantation, all mice received drinking water with analgesic medication (1.33 mg/mL metamizole, Zentiva Pharma GmbH, Frankfurt, Germany). On the day of surgery, mice were anesthetized with 2.5% (Vol) Isoflurane, then placed on a 37 °C thermoregulated mat (Thermo Mat Pro 20 W, Lucky reptile, 61202-HTP-20, Waltham, MA, USA); then, the skin was carefully disinfected with 70% (*v*/*v*) ethanol. Using the left flank incision as reported elsewhere [15], the pancreas was exposed, and the cell suspension was quickly injected into the body of the pancreas (see Supplementary Figure S1B). Thereafter, the pancreas was placed back in position, and the muscle and skin was sealed by a 3-knot surgical tie with resorbable (Ethibond 5/0, article number 6661H (Ethicon (J&J), Linz, Austria)) and non-resorbable (VicrylPlus 5/0 RB2 (45), article number VCP9982H (Ethicon)) sutures, respectively. Analgesia continued for a further 3 days after surgery. Mice were controlled 2–3 times per week for welfare and tumor formation. Considering that the doubling time of the cells is approximately 56 h, the tumors were expected to be detectable by imaging as from the fourth week post implantation.

### 4.4. Monitoring the Orthotopic Pancreatic Tumor Growth by Ultrasound (US) Imaging

An ultrasound system (Vevo700, VisualSonics, Toronto, ON, Canada) was used to monitor tumor growth. This was equipped with the thermoregulated imaging stage, including anesthesia supply and mouse nose cone, ultrasonic transducers (30 MHz), isoflurane anesthesia vaporizers/distributors (VetEquip, KF Technology Srl, Berlin, Germany) with vacuum exhaust supply for purging of exhaust gases, and an anesthesia induction chamber equipped with an exhaust gas purging exit. Further consumables used were eye ointment (Bepanthen, PZN: 01578675, Bayer Vital GmbH, Leverkusen, Germany) to prevent dryness, Transpore hypoallergenic surgical tape (3M Deutschland GmbH, Neuss, Germany) for fixing mice extremities on imaging stage, ultrasound transmission gel (Ref 01-50, Parker Laboratories Inc., Fairfield, NJ, USA), and Kimberly tissue wipes (Carl Roth GmbH +Co KG, Karlsruhe, Germany).

For imaging, mice were anesthetized in a sealed anesthesia induction chamber, with 2.5% (Vol) isoflurane in oxygen then a thin film of bepanthen eye ointment was applied. The mice were controlled for the initiation of anesthesia via overall movements and respiratory movements, and the depth of anesthesia was confirmed by the footpad reflex control. Sufficiently anesthetized mice were transferred from the induction chamber to the pre-warmed imaging stage equipped with a mouth cone with access to anesthesia. Moreover, 2.0% (Vol) isoflurane anesthesia delivered to the imaging stage was maintained throughout imaging. Depending on the area of interest for imaging, the animal was placed in a suitable position on the heated imaging stage with the nose placed inside the anesthesia nose cone. Mice extremities were fixed with low adhesive tape to circumvent the experimenter-induced movement of the animal during imaging (Figure 1). To monitor the tumor growth and progression from several perspectives, the mice were imaged first in the supine, then side positions. In the supine position, the visualization of the liver, stomach, duodenum, and infiltrating tumor growth towards these organs was possible. As a result, the ultrasound transducer was moved over the abdomen of the animal starting from the sternum (Figure 1A, line 1) to identify the liver, then navigating towards the stomach and duodenum in the direction of the mouse posterior (Figure 1A, line 2). After imaging the supine position, the mice were turned to the side position, and a generous amount of ultrasound gel was applied over the entire left side of the mouse (Figure 1B).

Imaging from the side position enables the visualization of the primary tumor within the pancreas, the spleen, kidney, and tumor growth towards these surrounding organs. For this, the transducer was placed on the animal orthogonal to the plane of the imaging platform and carefully navigated down to locate the spleen (cross section), then moved towards the posterior of the mouse (Figure 1B, line 1 towards line 2). Therefore, the underlying pancreas and tumor were located. Scanning through the whole tumor from here enabled the measurement of the tumor diameter and width. Since the Vevo700 used was not equipped with a tumor volume determination module, the determined tumor length and width was used to estimate the tumor volume according to [56], using the following formula:

$$V=\frac{\pi }{6}f{\left(Length\times width\right)}^{3/2}$$

where *f* = 1.58.

### 4.5. Whole Body Fluorescence Imaging of Mice

In few of the mice bearing Panc-1-fl tumors, fluorescence imaging tomography (FLIT) images of the whole body were acquired on an IVIS-Spectrum system (PerkinElmer Inc., Shelton, CT, USA), based on the mkate2 fluorescence (absorption/emission: 588 nm/633 nm). To pinpoint the metastatic spread and the location of the tumors, the situs of dissected mice were imaged on a Maestro^TM^ fluorescence imaging system (Cri-INTAS, Woburn, UK) using the yellow filterset (activation: 575–605 nm Emission: 645 nm longpass) and capturing fluorescence signals from 630 to 850 nm in 10 nm steps.

### 4.6. Histological Analysis of Tumor Fibroblast and Collagen Levels

The immunohistochemical analysis of naïve pancreatic stellate cells, based on Vitamin A content, and activated tumor-associated fibroblasts, based on their expression of alpha-smooth muscle actin (αSMA) and fibroblast activation protein (FAP), were implemented together with collagen stain to validate tumor progression-related fibrosis. Vitamin A was immuno-detected using the biotinylated rabbit anti-Vitamin-A polyclonal antibody (from antibodies online.com, catalog number ABIN1175920). Mature tumor fibroblasts were stained with a rabbit anti-human, -mouse, and -rat alpha-smooth muscle actin monoclonal antibody D4K9N (Cell Signaling, Leiden, The Netherlands, mAb#19245) or a rabbit anti-human, -mouse, and -rat FAP polyclonal antibody (Thermo Scientific, Schwerte, Germany, PA5-99313). Briefly, dewaxed sections were subjected to antigen retrieval (25 min heating at sub-boiling temperature in 10 mM citrate buffer pH 6.0), subsequent cooling, washing with Tris buffered saline containing 0.1% (*v*/*v*) Tween-20 (TBS-T), and blocking with avidin and biotin (Dako, Glostrup, Germany), according to the product manual. The sections were washed with TBS-T and immuno-probed overnight at 4 °C with the respective antibodies (diluted in Dako antibody diluent at 1:100 for the anti-Vitamin-A pAb, and 1:800 for the alpha-SMA-mAb and 1:200 for the FAP-pAb). After two wash steps of 5 min at room temperature (RT), slices probed with the αSMA or FAP antibodies were incubated for 45 min at RT with a goat anti-rabbit biotinylated secondary antibody (Dianova GmbH, Hamburg, Germany, 1:250 in antibody diluent). Then, all the sections were washed briefly with TBS-T, incubated for 45 min with Streptavidin Alkaline Phosphatase (Southern Biotech, Birmingham, AL, USA, Catalog number 7100-04), and subsequently washed and detected with a Chromogen (Dako, Glostrup, Germany). Nuclei were counter-stained with hematoxylin (Sigma-Aldrich, Karlsruhe, Germany). The sections were then mounted with Faramount (Dako, Glostrup, Germany) and cover-slipped for microscopy.

Collagen fiber was stained with Picro-Sirius red solution. Dewaxed sections were rinsed with water and incubated for 1 h in Picro-Sirius red solution (Morphisto, Frankfurt, Germany). The sections were briefly rinsed with 10 mM hydrochloric (HCl) acid and incubated for 2 min in fresh HCL. Gradual dehydration by brief incubation (1 min) in absolute ethanol and 2 min in xylene followed. Finally, the sections were mounted with PERTEX^®^ mounting medium (VWR International GmbH, Dresden, Germany) cover-slipped and air-dried for microscopy.

Microscopic images of the whole tumors at 4× magnification and larger extracts at a 20× or 40× magnification were captured on the Keyence BZ-X800 microscope (Keyence Deutschland GmbH, Neu Issenburg, Germany). The semi-quantitative estimation of the number of stained cells according to bio-protocol [57] or collagen fibers [58] within the captured images was conducted using the Fiji-ImageJ software Version 1.8 (NIH, Bethesda, MD, USA). For this, we implemented tumor tissues of 1–4 mice and 4–6 images from the tumor periphery and center of each section.

### 4.7. Statistical Analysis

The differences in the number of stained fibroblastic cells or the level of collagen at the investigated tumor growth time points (all determined within the captured images using the Fiji-ImageJ software, see above) were deduced by unpaired *t*-test with Welch correction using the Prism 9 software. Differences resulting in *p* < 0.05 were considered statistically significant.

## 5. Conclusions

Using standardized human cancer cell-based orthotopic pancreatic cancer models in mice; ultrasound imaging and histochemical analysis of naïve pancreatic stellate cells; mature fibroblasts (myCAF and iCAF); and collagen, we highlight some morphological and molecular features that could guide the preclinical definition of orthotopic pancreatic tumor progression. Based on the observations and the tumor progression-based parameters, we provide suggestions for the future design, monitoring, and selection of preclinical pancreatic cancer models for therapy testing purposes. We are convinced that the implementation of these suggestions and modifications thereof will improve the preclinical testing and selection of suitable therapeutics for the management of pancreatic adenocarcinomas and other orthotopic tumor mouse models, such as ovarian and colon carcinomas in the future.

## Figures and Tables

**Table 1:** Overall observations and suggested progression-dependent tumor parameters.

Tumor Progression *	Estimated Time Post Induction	US Detection of Primary Tumor		US Detection of Infiltration		Histological Validations			
		Side Position	Supine Position	Side Position	Supine Position	PSCs Vit-A	myCAF aSMA	iCAFs FAP (Stroma)	Fibrosis Collagen
**Early**	2–4 weeks	Small tumor secluded in pancreas	No peculiarity	No peculiarity	No peculiarity	Very low <5% oval cells	>20%	−/+	≤20%
**Advanced**	5–7 weeks	Large tumor	Growth towards gastric lobe/omentum	Growth towards gastric lobe/omentum	Omental infiltration	Low, <10% mix of oval/elongated cells	≥40%	+	≥40%
**Late**	≥7 weeks	-Very large tumor -Metastasis in surrounding organs	-Pressure on stomach -Tumor in omentum	-Metastasis in surrounding organs, e.g., spleen	-Pressure on stomach -Omental/or stomach wall infiltration -Metastasis	>10% Mostly elongated	>40%	+++	> 60%

* The suggested progression-dependent tumor parameters are deduced from the Panc-1-fl and BxPC-3 cells implanted in the presence of small amounts of a supportive Matrigel^®^ matrix that helps the cells to initially be secluded within the pancreas after implantation. The duration of growth and appearance of the features described for the growth stages vary depending on the aggressiveness of the cell line. It is noteworthy that image-based morphological findings considered the Panc-1-fl predominantly since the BxPC-3 metastasized earlier, and mice were sacrificed at 8 weeks post implantation. Number of mice and tissues considered: Panc-1-fl (20 mice for morphology and 9 mice for histology) and BxPC-3 (10 mice for morphology and 4 mice for histology). US: ultrasound.

## Data Availability

The original contributions presented in the study are included in part in the article/Supplementary Material, and further data shall be available upon request.

## Supplementary Materials

The supporting information can be downloaded at: https://www.mdpi.com/article/10.3390/ijms25115619/s1.
