# Supplementary material for: Identifying the Morphological and Molecular Features of a Cell-Based Orthotopic Pancreatic Cancer Mouse Model during Growth over Time
Source: Int J Mol Sci. 2024 May 22;25(11):5619. doi: 10.3390/ijms25115619 (PMC11171605; doi:10.3390/ijms25115619)
Supplement: Supplementary file 1 [file ijms-25-05619-s001.zip › Supplementary Figures R2-ijms-2986733.pdf]

International Journal of *Molecular Sciences*

Article, Supplementary material

Identifying the Morphological and Molecular Features of a Cell-Based Orthotopic Pancreatic Cancer Mouse Model during Growth over Time

Felista L. Tansi [1,*], Andrea Schrepper [2], Michael Schwarzer [2], Ulf Teichgräber [3] and Ingrid Hilger [1,*]

[1] Experimental Radiology, Institute of Diagnostic and Interventional Radiology, Jena University Hospital, Friedrich Schiller University Jena, Am Klinikum 1, 07747 Jena, Germany
[2] Department of Cardiothoracic Surgery, Jena University Hospital, Friedrich Schiller University Jena, Am Klinikum 1, 07747 Jena, Germany
[3] Institute of Diagnostic and Interventional Radiology, Jena University Hospital, Friedrich Schiller University Jena, Am Klinikum 1, 07747 Jena, Germany
* Correspondence: felista.tansi@med.uni-jena.de (F.L.T.); ingrid.hilger@med.uni-jena.de (I.H.); Tel.: +49-3641-9324993 (F.L.T.); +49-3641-9325921 (I.H.)

Supplementary Figure S1: Photographic and schematic illustration of the location of the omentum and pancreas in the mouse.

The figure illustrates the anatomical locations of omental fat in the mouse. A.) Photograph of a dissected healthy mouse exposing the situs with omental fat (Om) in the side view and splenoportal fat (SPF) seen after the spleen is pulled with forceps (back view). A better understanding of the anatomy can be got when comparing the schematic drawing of the macroscopic anatomy of mouse pancreas and surrounding organs according to Dolenšek *et al.* [1] under consideration of the omental fat according to observations of the mouse situs and literature [2], as shown in Figure 6B of the main article. B) Representative photograph of excised pancreas (either empty (left image) or filled by injection of 200 µl PBS, (right image)), with surrounding organs (stomach, duodenum, spleen and omental fat). As seen in the image, the omentum (Om*) extends from the edge of the splenic lobe to the edge of the gastric lobe and is not filled even after injection of 200 µl PBS.

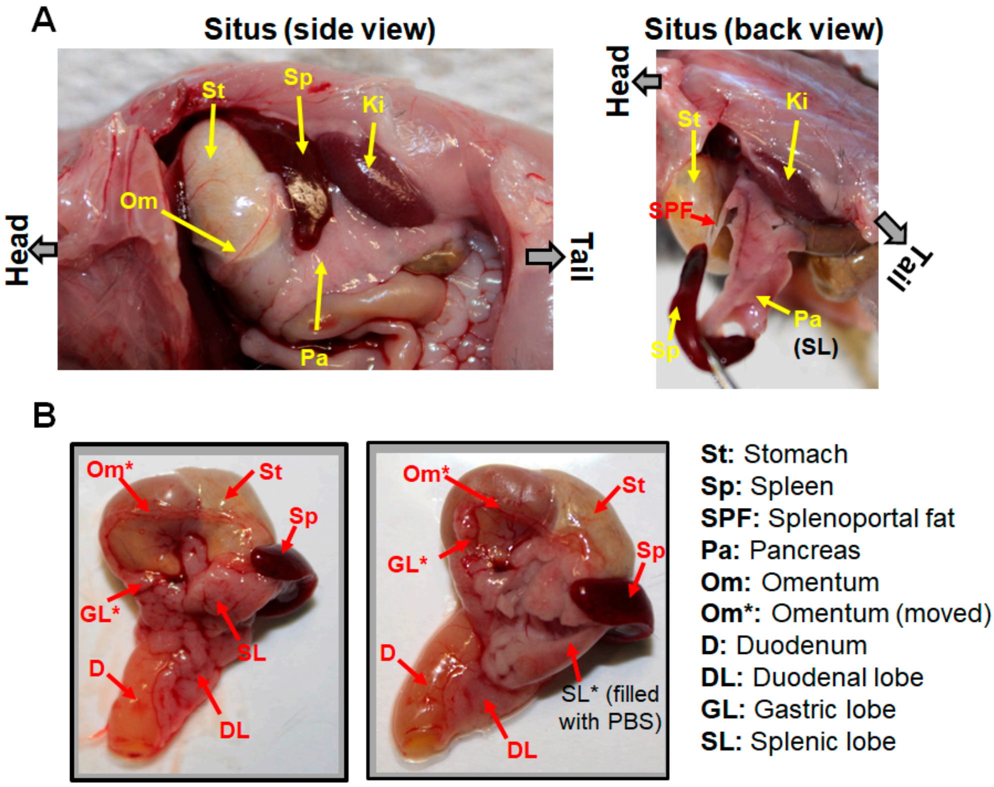

Figure S1: Validation of the anatomy of the pancreas, omental fat and splenoportal fat in the mouse.

Supplementary Figure S2: The pancreatic cancer cell line BxPC-3 reveals a faster disease progression and metastasis formation than Panc-1 cells after orthotopic implantation in mice.

Whereas the Panc-1-fl cells developed palpable primary tumors within the mice pancreas as from 3 weeks post implantation in 2% (v/v) Matrigel® matrix in HBSS, the BxPC-3 based tumors rapidly spread and infiltrated the stomach wall and other organs post implantation (Supplementary Figure S2). This indicates that the use of the Matrigel® matrix at low concentrations does not alter the aggressiveness and progression of different tumor models. However, this observation suggests that the concentration of the Matrigel® matrix should be increased for such aggressive models, in order to enable the growth and seclusion of a primary tumor during the first few weeks of implantation. Furthermore, it reiterates the importance of monitoring unique features that expose the advancement of the orthotopic tumor models, rather than only considering the time point post implantation, as is often the case in most research reports. Hence, imaging the growth and progression from when a secluded tumor is formed as reported herewith reveals the developmental stages of the tumors, which can serve as useful tools for selection of particular stages of interest for preclinical therapeutic efficacy assessment.

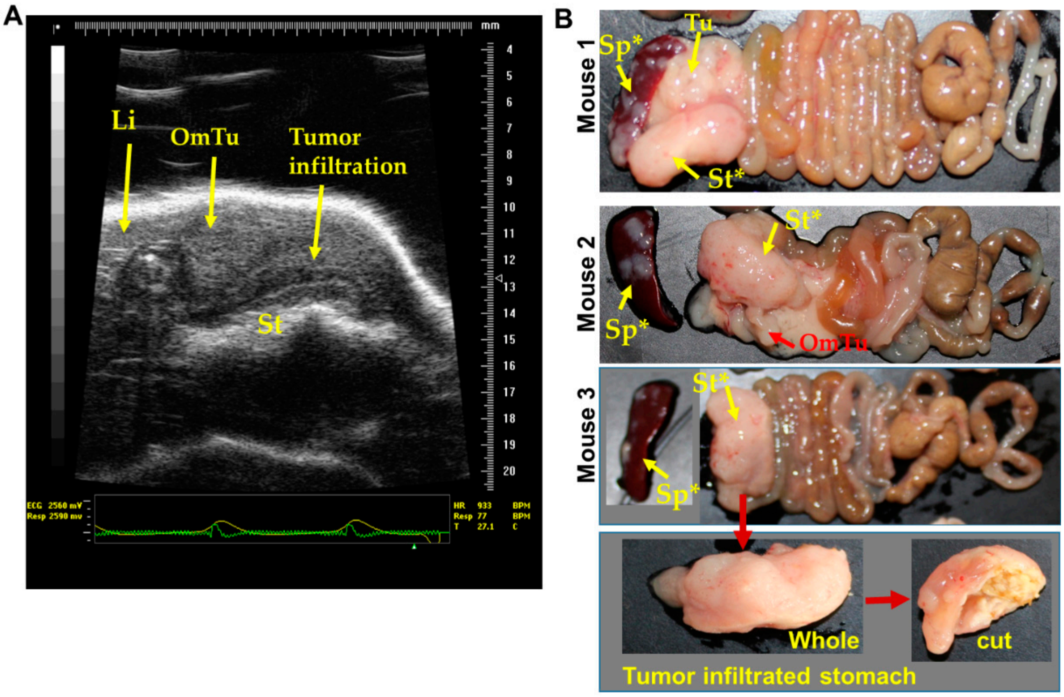

Figure S2: Demonstrations of the aggressive nature of the BxPC-3 tumor model. A) Representative ultrasound image of a mouse in the supine position showing the distorted stomach and infiltrated stomach wall at 7 weeks after tumor induction. B) Examples of organs isolated from 3 mice that revealed tumor infiltration of the stomach wall (St*) and metastatic lesions of the spleen (Sp*). These were excised 8 weeks after tumor induction).

Supplementary Figure S3: Blood values of mice bearing the Panc-1 and the aggressive BxPC-3 tumor models do not differ significantly.

Despite the aggressive nature of the BxPC-3 tumors, the blood values from mice bearing the BxPC-3 tumor models showed no significant difference compared to blood from mice that were induced with the Panc-1 based tumor models (Supplementary Figure S3). Platelets usually increase during metastatic cell circulation in blood, since they protect the tumor cells from natural killer cells during their dissemination to distant organs. Though there was a slight increase in platelets (PLT) in BxPC-3 as compared to Panc-1 bearing mice blood, this was not significantly higher.

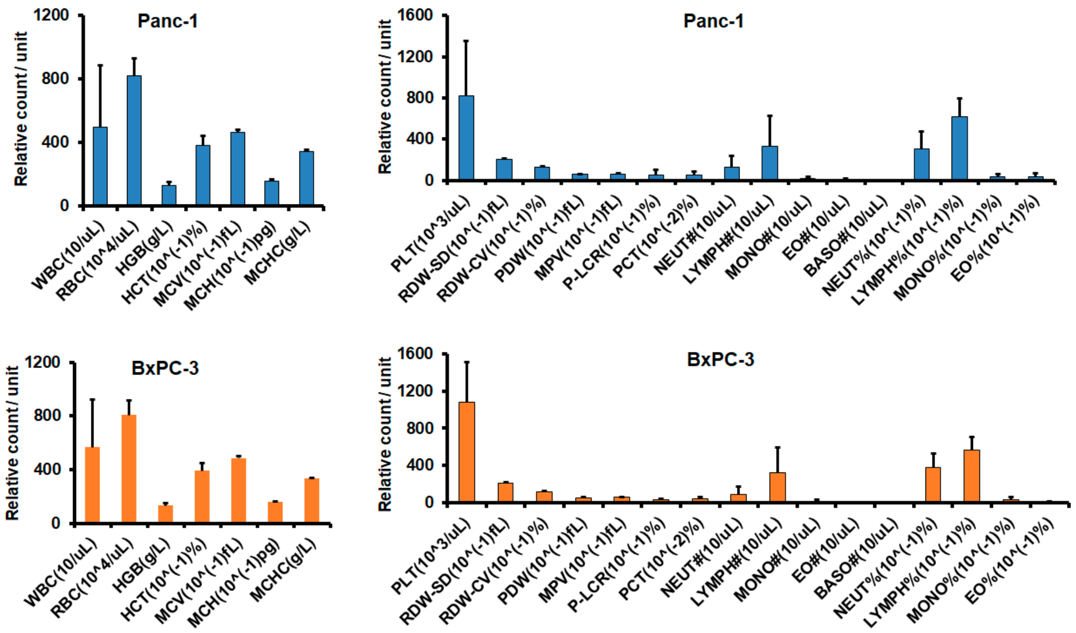

Figure S3: Blood values of mice bearing the Panc-1 and the aggressive BxPC-3 tumor models. Although the BxPC-3 tumor models revealed a more aggressive progression and infiltration of the stomach wall with increased metastasis formation, the blood values from the mice are almost identical to those from mice bearing Panc-1 tumors. Each bar represents the values of 8 mice / tumor model and the standard deviation. Time point of blood collection is 8 weeks for BxPC-3 and about 10 weeks for the Panc-1.

Supplementary Figure S4: Validation of tumor progression related levels of collagen fibers in tumors

Histological slices of tumors excised together with surrounding pancreas and spleen (week 4) or additionally with the stomach and intestinal tract were stained with picrosirius red for collagen fiber detection as described in the main article. Whole mount images were acquired with the Keyence BZ-X800 microscope at 4x magnification. The collagen fiber stain in the tumors become more evident over time during progression

from week 4 to week 10 post induction (Figure S4A). Interestingly the level of collagen is significantly lower (**P < 0.002) than the level of myofibroblastic cancer associated fibroblasts (myCAF, [3]) detected by αSMA stain at 4 weeks after induction and significantly higher (***P < 0.0005) at 10 weeks after induction, whereas there is no difference at 8 weeks post tumor induction (Figure S4B). This suggests the infiltration of tumors by fibroblasts to a plateau level during tumor progression, and the persistent production of collagen fiber thereafter, till later growth stages.

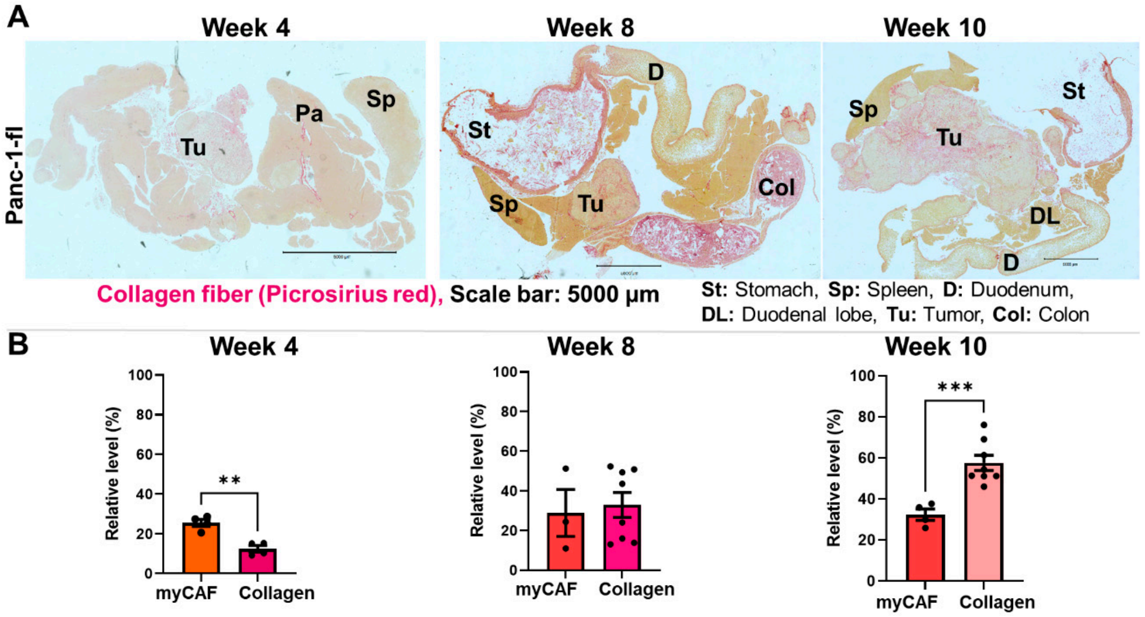

Figure S4: Histological analysis of collagen levels. **A)** Whole section mount light microscopic images of Panc-1-fl orthotopic tumors and surrounding organs showing picrosirius red stained collagen fiber. An increase in collagen fibers in the tumors is evident from week 4 through week 10. **B)** Semiquantitative comparison of tumor myofibroblasts (SMA-alpha positive, myCAFs) versus tumor collagen levels. Each bar represents the relative area of tumor with collagen or the percentage of cells expressing SMA-alpha within the slices of 1-2 animals. Error bars represent standard error of means (SEM.). **P < 0.002, ***p < 0.0005 as determined by unpaired t test with Welch correction.

Supplementary Figure S5: Whole mount image of a BxPC-3 tumor model

The figure shows a whole mount image of a BxPC-3 tumor model stained to detect pancreatic stellate cells via vitamin A, myofibroblasts by αSMA, and collagen fibers using picrosirius red. It must be noted that this image is an example and not representative of all BxPC-3 tumors, as the BxPC-3 orthotopic model grew rapidly and ununiformly.

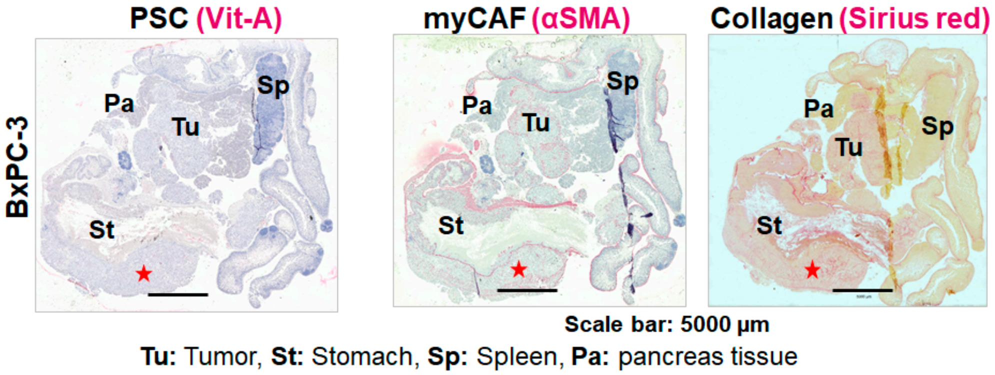

Figure S5: Whole section mount images of the BxPC-3 tumor model at week 8, showing the primary tumor (Tu), infiltrated stomach wall (red star), and surrounding pancreas (Pa), spleen (SP) and the intestines. The histological slice was stained for pancreatic stellate cells (PSCs), myofibroblasts (myCAF) and collagen as indicated by vitamin A (Vit A), alpha-smooth muscle actin (aSMA), and picrosirius red, respectively.

Supplementary Figure S6: Relative analysis of iCAFs in tumors

The figure shows the images of FAP stained Panc-1-fl and BxPC-3 tumor slices and the bar diagram demonstrating the relative increase in number of iCAFs within tumors, deduced from 5 arbitrary unit areas in the periphery and center of tumors respectively. The chalkley system was used, whereby all iCAFs (tiny elongated stromal cells with FAP signals) found within the chalkley ring were counted.

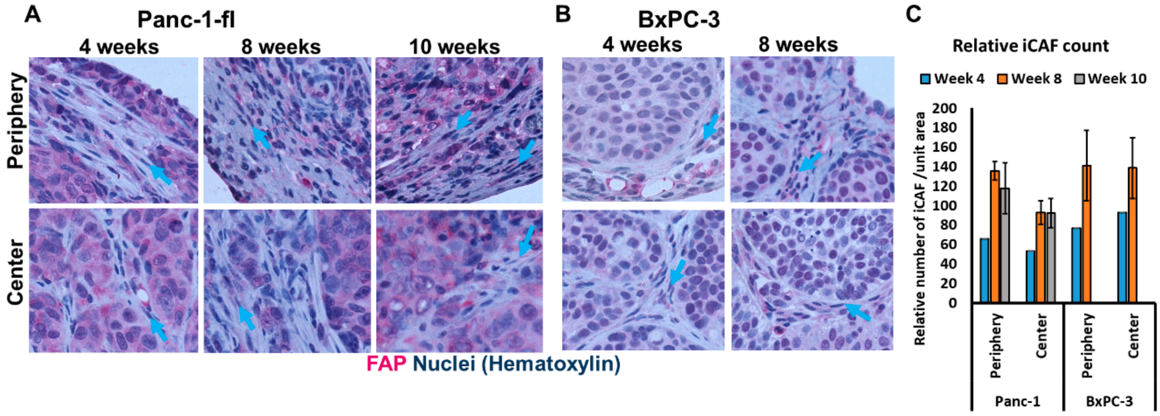

Figure S6: Relative analysis of iCAFs in tumors. Images of FAP stained slices of A) Panc-1-fl and B) BxPC-3 tumors showing the tiny elongated inflammartory fibroblasts in the stroma (blue arrows). C) Bar plot of the relative number of FAP-positive iCAFs counted within 5 unit areas (based on chalkley system) in the periphery and center of the tumors. N=1, 2 and 4 mice for week 4, week 8 and week 10 tumors, respectively.

References

1. Dolenšek, J.; Rupnik, M.S.; Stožer, A. Structural similarities and differences between the human and the mouse pancreas. *Islets* **2015**, *7*, e1024405, doi:10.1080/19382014.2015.1024405.
2. Krishnan, V.; Clark, R.; Chekmareva, M.; Johnson, A.; George, S.; Shaw, P.; Seewaldt, V.; Rinker-Schaeffer, C. In Vivo and Ex Vivo Approaches to Study Ovarian Cancer Metastatic Colonization of Milky Spot Structures in Peritoneal Adipose. *J Vis Exp* **2015**, 10.3791/52721, e52721, doi:10.3791/52721.

3. Öhlund, D.; Handly-Santana, A.; Biffi, G.; Elyada, E.; Almeida, A.S.; Ponz-Sarvise, M.; Corbo, V.; Oni, T.E.; Hearn, S.A.; Lee, E.J., et al. Distinct populations of inflammatory fibroblasts and myofibroblasts in pancreatic cancer. *J Exp Med* **2017**, *214*, 579-596, doi:10.1084/jem.20162024.
