# Supplementary material for: Identifying the Morphological and Molecular Features of a Cell-Based Orthotopic Pancreatic Cancer Mouse Model during Growth over Time
Source: Int J Mol Sci. 2024 May 22;25(11):5619. doi: 10.3390/ijms25115619 (PMC11171605; doi:10.3390/ijms25115619)
Supplement: Supplementary file 1 [file ijms-25-05619-s001.zip › Supplementary Video S1.pptx]

## Slide 1
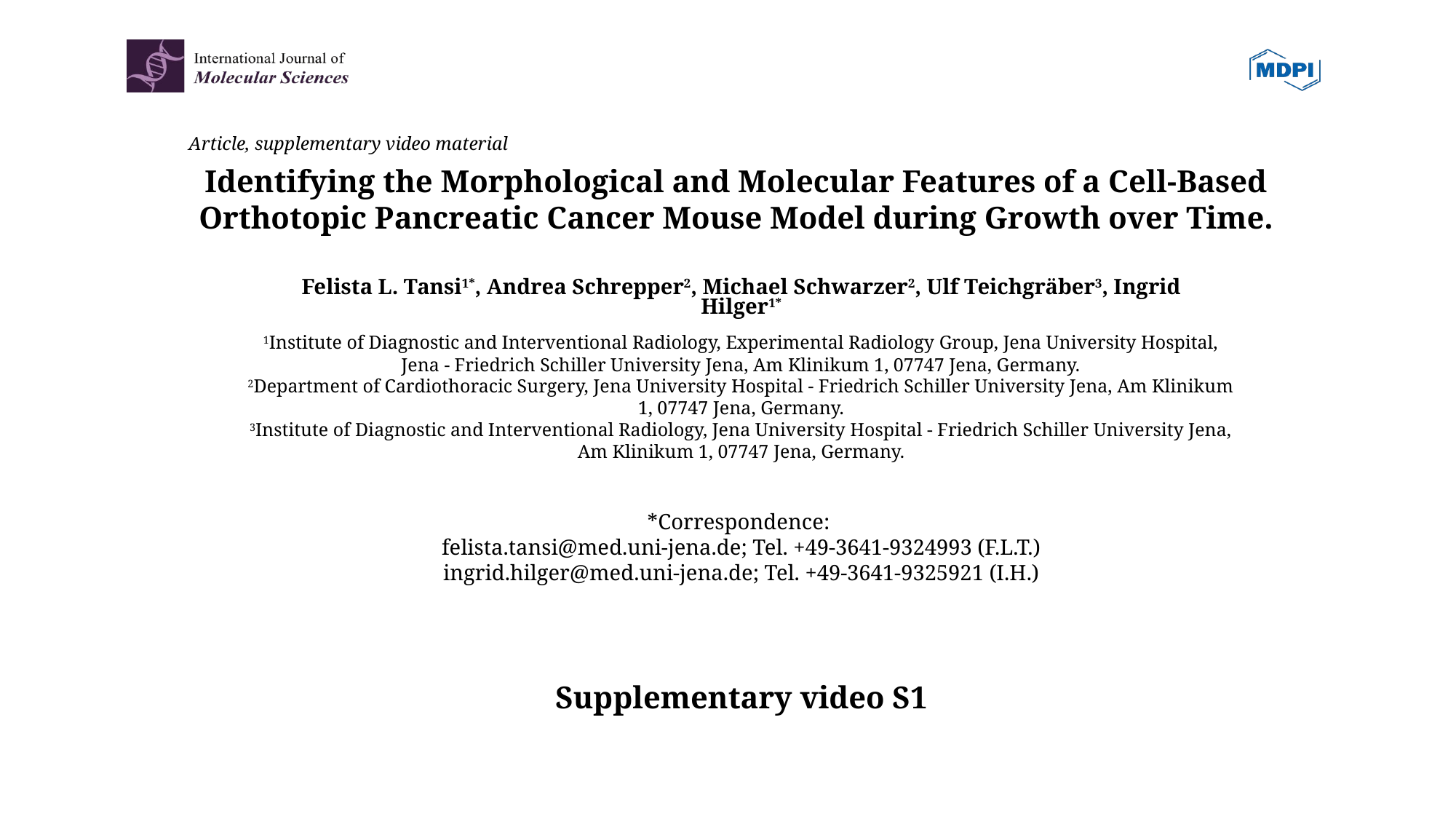

| | | |
| --- | --- | --- |
Article, supplementary video material
Identifying the Morphological and Molecular Features of a Cell-Based Orthotopic Pancreatic Cancer Mouse Model during Growth over Time.
Felista L. Tansi1*, Andrea Schrepper2, Michael Schwarzer2, Ulf Teichgräber3, Ingrid Hilger1*
1Institute of Diagnostic and Interventional Radiology, Experimental Radiology Group, Jena University Hospital, Jena - Friedrich Schiller University Jena, Am Klinikum 1, 07747 Jena, Germany.
2Department of Cardiothoracic Surgery, Jena University Hospital - Friedrich Schiller University Jena, Am Klinikum 1, 07747 Jena, Germany.
3Institute of Diagnostic and Interventional Radiology, Jena University Hospital - Friedrich Schiller University Jena, Am Klinikum 1, 07747 Jena, Germany.
*Correspondence:
felista.tansi@med.uni-jena.de; Tel. +49-3641-9324993 (F.L.T.)
ingrid.hilger@med.uni-jena.de; Tel. +49-3641-9325921 (I.H.)
Supplementary video S1

## Slide 2
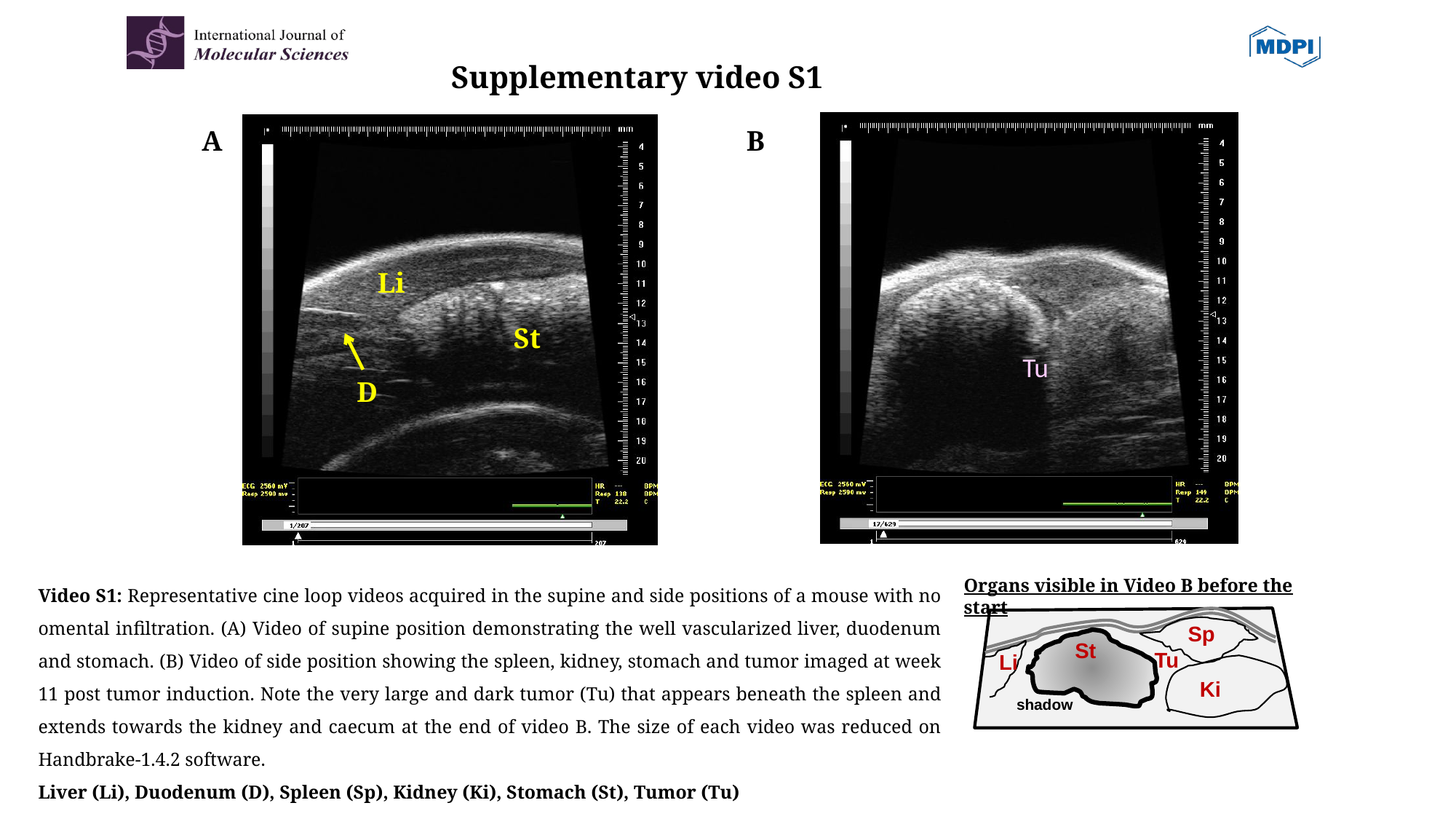

Supplementary video S1
A
B
Li
St
Tu
D
Video S1: Representative cine loop videos acquired in the supine and side positions of a mouse with no omental infiltration. (A) Video of supine position demonstrating the well vascularized liver, duodenum and stomach. (B) Video of side position showing the spleen, kidney, stomach and tumor imaged at week 11 post tumor induction. Note the very large and dark tumor (Tu) that appears beneath the spleen and extends towards the kidney and caecum at the end of video B. The size of each video was reduced on Handbrake-1.4.2 software.
Liver (Li), Duodenum (D), Spleen (Sp), Kidney (Ki), Stomach (St), Tumor (Tu)
Organs visible in Video B before the start
Sp
St
Li
Ki
Tu
shadow
