# Supplementary material for: Identifying the Morphological and Molecular Features of a Cell-Based Orthotopic Pancreatic Cancer Mouse Model during Growth over Time
Source: Int J Mol Sci. 2024 May 22;25(11):5619. doi: 10.3390/ijms25115619 (PMC11171605; doi:10.3390/ijms25115619)
Supplement: Supplementary file 1 [file ijms-25-05619-s001.zip › Supplementary Video S2.pptx]

## Slide 1
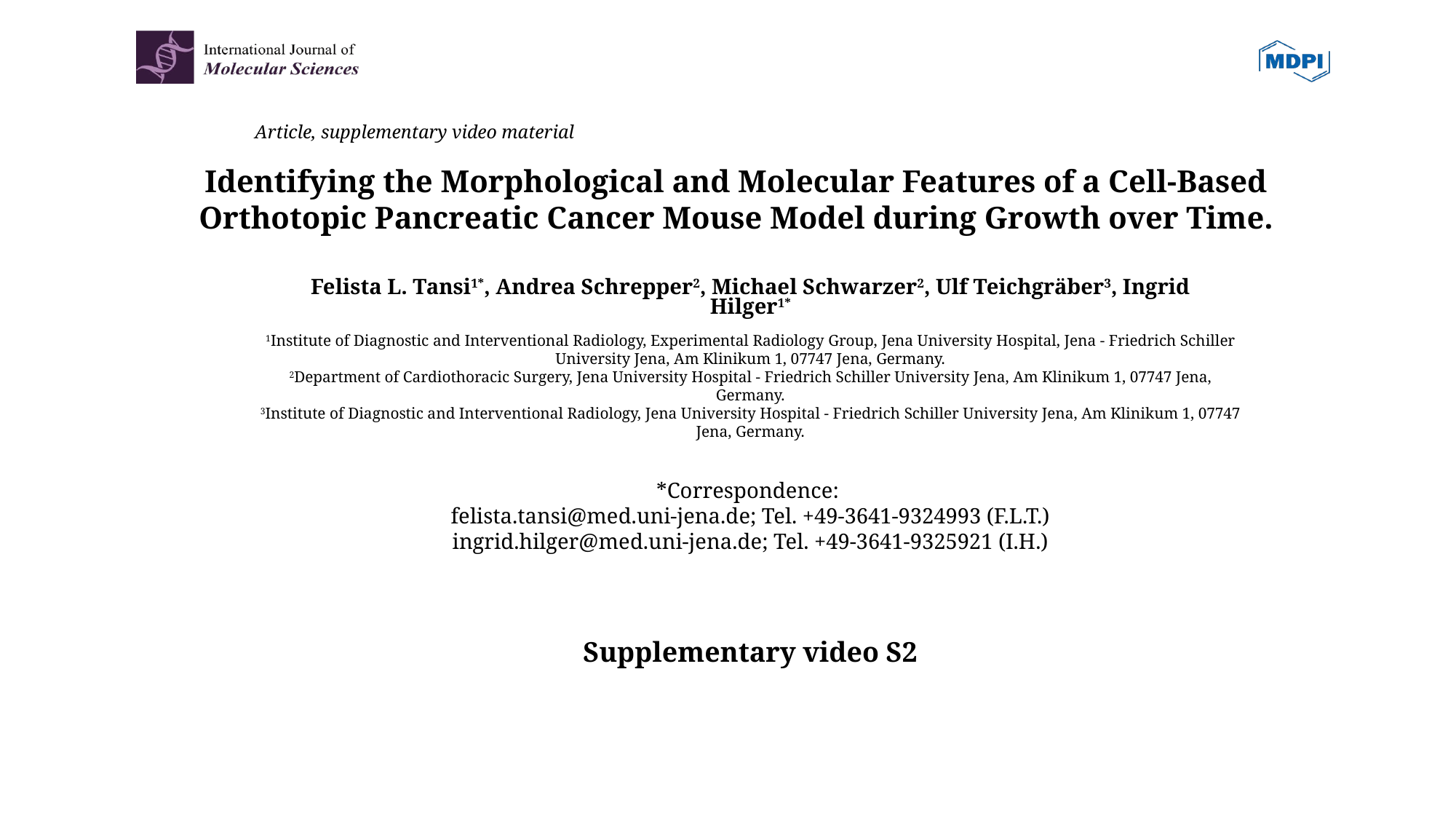

Article, supplementary video material
Identifying the Morphological and Molecular Features of a Cell-Based Orthotopic Pancreatic Cancer Mouse Model during Growth over Time.
Felista L. Tansi1*, Andrea Schrepper2, Michael Schwarzer2, Ulf Teichgräber3, Ingrid Hilger1*
1Institute of Diagnostic and Interventional Radiology, Experimental Radiology Group, Jena University Hospital, Jena - Friedrich Schiller University Jena, Am Klinikum 1, 07747 Jena, Germany.
2Department of Cardiothoracic Surgery, Jena University Hospital - Friedrich Schiller University Jena, Am Klinikum 1, 07747 Jena, Germany.
3Institute of Diagnostic and Interventional Radiology, Jena University Hospital - Friedrich Schiller University Jena, Am Klinikum 1, 07747 Jena, Germany.
*Correspondence:
felista.tansi@med.uni-jena.de; Tel. +49-3641-9324993 (F.L.T.)
ingrid.hilger@med.uni-jena.de; Tel. +49-3641-9325921 (I.H.)
Supplementary video S2

## Slide 2
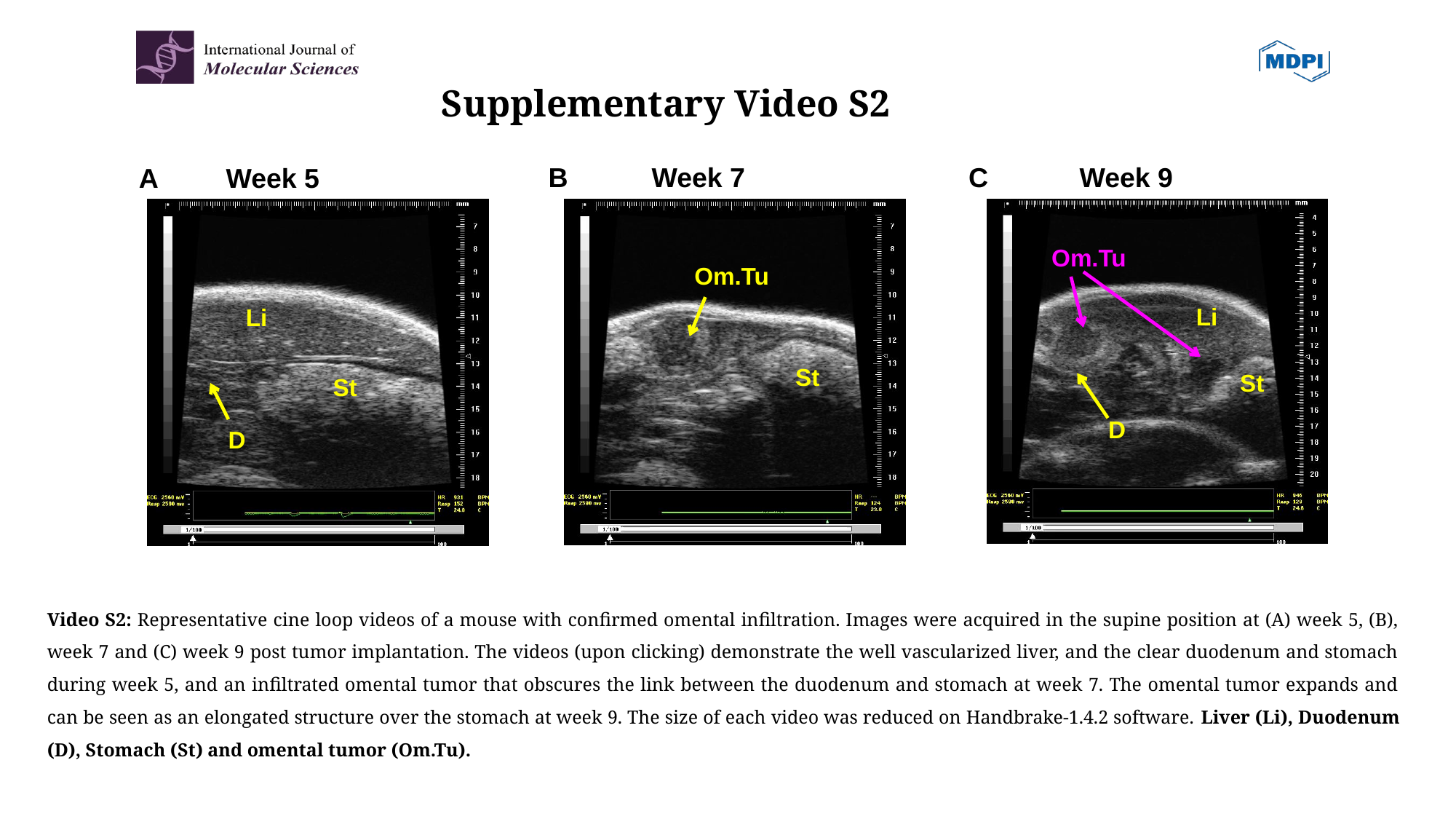

Supplementary Video S2
B Week 7
C Week 9
A Week 5
Om.Tu
Om.Tu
Li
Li
St
St
St
D
D
Video S2: Representative cine loop videos of a mouse with confirmed omental infiltration. Images were acquired in the supine position at (A) week 5, (B), week 7 and (C) week 9 post tumor implantation. The videos (upon clicking) demonstrate the well vascularized liver, and the clear duodenum and stomach during week 5, and an infiltrated omental tumor that obscures the link between the duodenum and stomach at week 7. The omental tumor expands and can be seen as an elongated structure over the stomach at week 9. The size of each video was reduced on Handbrake-1.4.2 software. Liver (Li), Duodenum (D), Stomach (St) and omental tumor (Om.Tu).
