# Supplementary material for: Identifying the Morphological and Molecular Features of a Cell-Based Orthotopic Pancreatic Cancer Mouse Model during Growth over Time
Source: Int J Mol Sci. 2024 May 22;25(11):5619. doi: 10.3390/ijms25115619 (PMC11171605; doi:10.3390/ijms25115619)
Supplement: Supplementary file 1 [file ijms-25-05619-s001.zip › Supplementary Video S3_BxPC-3.pptx]

## Slide 1
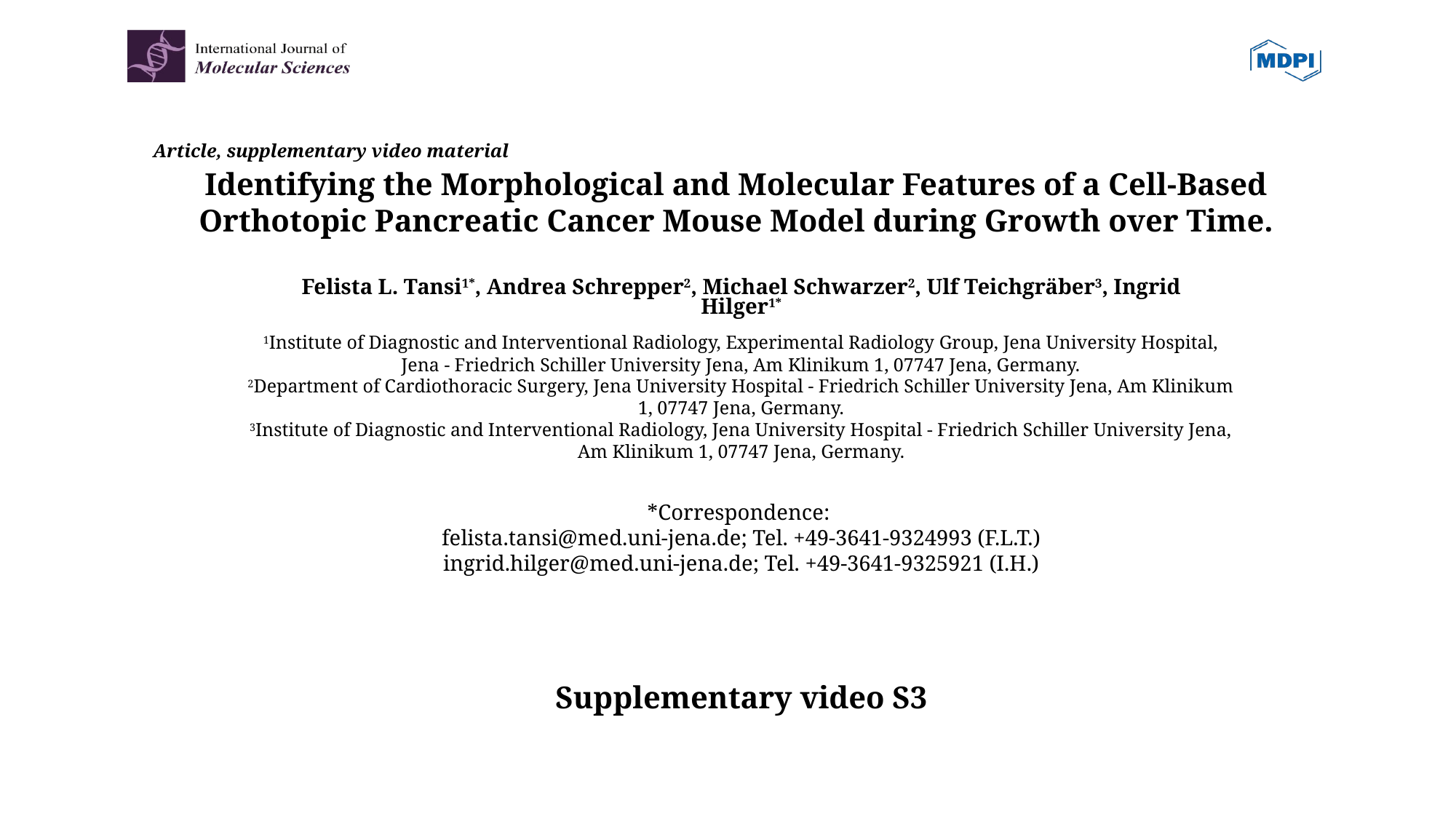

Article, supplementary video material
Identifying the Morphological and Molecular Features of a Cell-Based Orthotopic Pancreatic Cancer Mouse Model during Growth over Time.
Felista L. Tansi1*, Andrea Schrepper2, Michael Schwarzer2, Ulf Teichgräber3, Ingrid Hilger1*
1Institute of Diagnostic and Interventional Radiology, Experimental Radiology Group, Jena University Hospital, Jena - Friedrich Schiller University Jena, Am Klinikum 1, 07747 Jena, Germany.
2Department of Cardiothoracic Surgery, Jena University Hospital - Friedrich Schiller University Jena, Am Klinikum 1, 07747 Jena, Germany.
3Institute of Diagnostic and Interventional Radiology, Jena University Hospital - Friedrich Schiller University Jena, Am Klinikum 1, 07747 Jena, Germany.
*Correspondence:
felista.tansi@med.uni-jena.de; Tel. +49-3641-9324993 (F.L.T.)
ingrid.hilger@med.uni-jena.de; Tel. +49-3641-9325921 (I.H.)
Supplementary video S3

## Slide 2
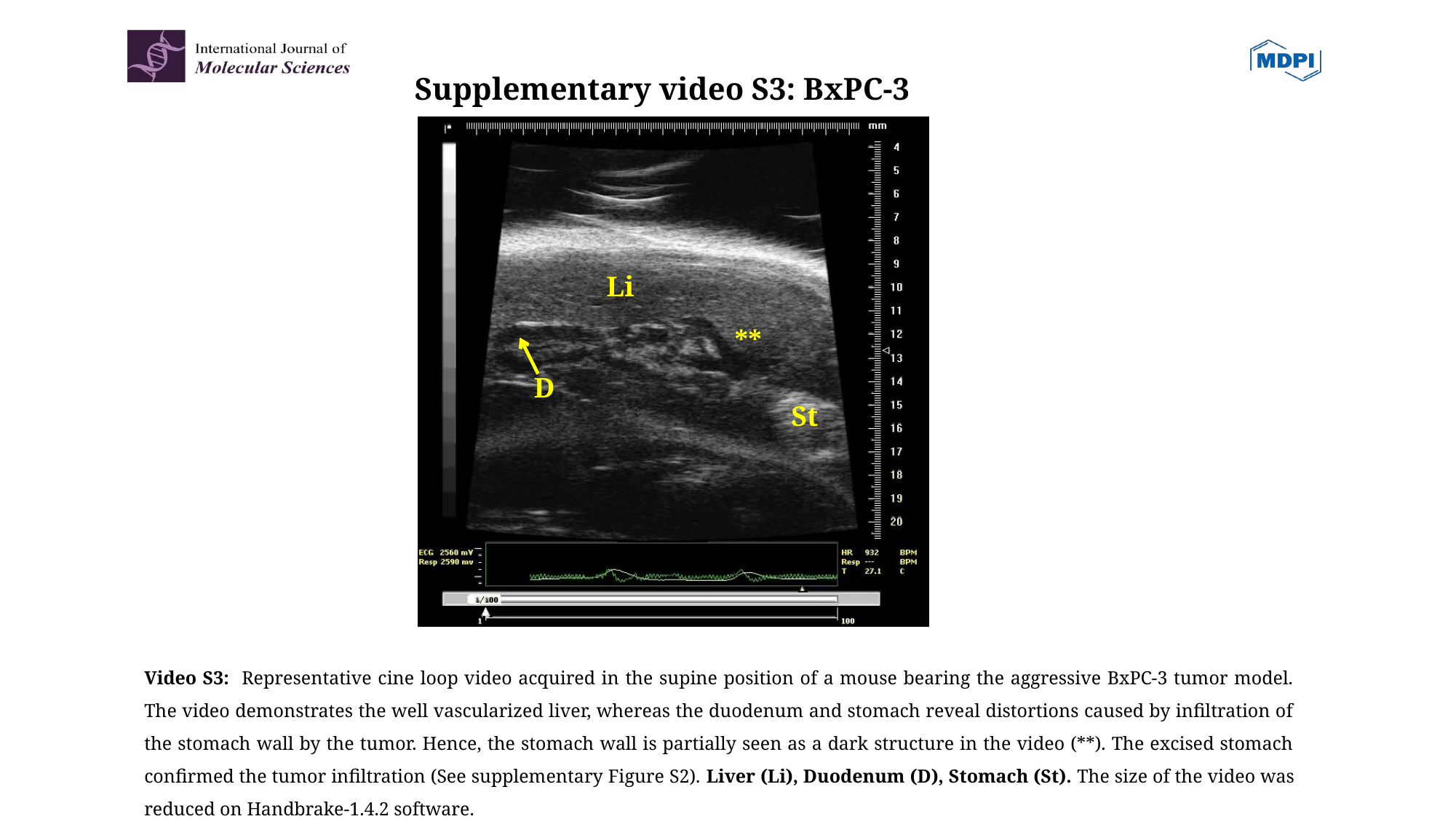

Supplementary video S3: BxPC-3
Li
**
D
St
Video S3: Representative cine loop video acquired in the supine position of a mouse bearing the aggressive BxPC-3 tumor model. The video demonstrates the well vascularized liver, whereas the duodenum and stomach reveal distortions caused by infiltration of the stomach wall by the tumor. Hence, the stomach wall is partially seen as a dark structure in the video (**). The excised stomach confirmed the tumor infiltration (See supplementary Figure S2). Liver (Li), Duodenum (D), Stomach (St). The size of the video was reduced on Handbrake-1.4.2 software.
